# Supplementary figures and images for: Informing policy via dynamic models: Cholera in Haiti
Source: PLoS Comput Biol. 2024 Apr 29;20(4):e1012032. doi: 10.1371/journal.pcbi.1012032 (PMC11081515; doi:10.1371/journal.pcbi.1012032)

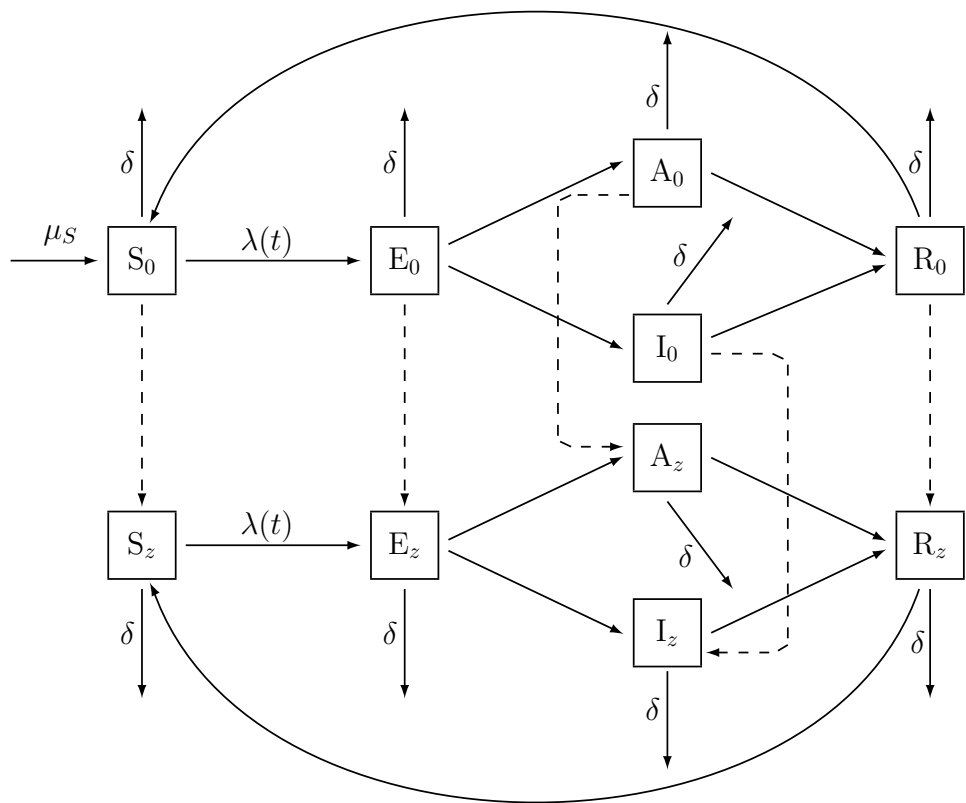

A flow diagram for the SEAIR model.

Supplement: S1 Fig — Flow chart representation of Model 1. (PDF) [file pcbi.1012032.s001.pdf]
