# Supplementary material for: Informing policy via dynamic models: Cholera in Haiti
Source: PLoS Comput Biol. 2024 Apr 29;20(4):e1012032. doi: 10.1371/journal.pcbi.1012032 (PMC11081515; doi:10.1371/journal.pcbi.1012032)
Supplement: S2 Fig — Flow chart representation of Model 2. (PDF) [file pcbi.1012032.s002.pdf]

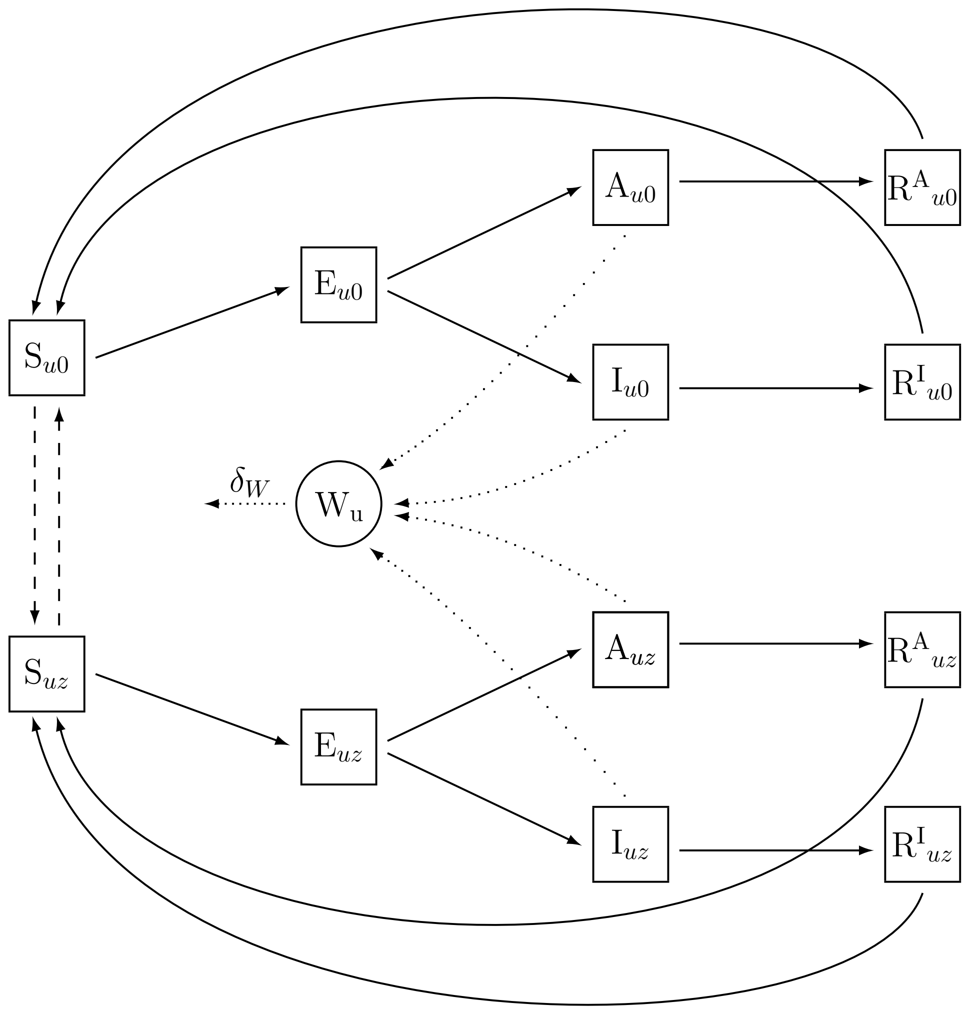

A flow diagram for the SEAIR model 2. This is a constant population model, there are no births/deaths. Vaccinations are assumed to only be given to susceptible individuals, and vaccine immunity wanes only with susceptible vaccinated individuals.
