# Supplementary material for: Informing policy via dynamic models: Cholera in Haiti
Source: PLoS Comput Biol. 2024 Apr 29;20(4):e1012032. doi: 10.1371/journal.pcbi.1012032 (PMC11081515; doi:10.1371/journal.pcbi.1012032)
Supplement: S3 Fig — Flow chart representation of Model 3. (PDF) [file pcbi.1012032.s003.pdf]

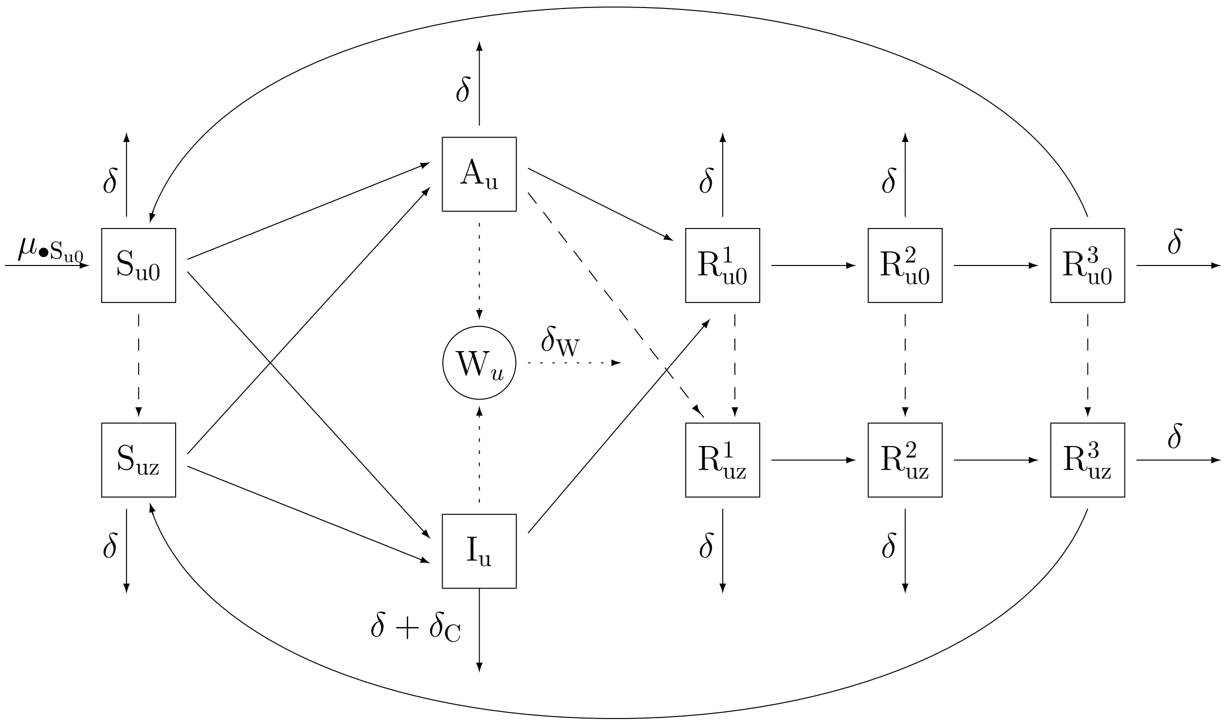

A flow diagram for the SAIR model 3. This model assumes a constant population while also including a mechanism for births/deaths; all deaths are balanced by births into the unvaccinated susceptible compartment, so the birth rate $\mu_{\bullet S_{u0}}$ corresponds to the sum total deaths from the remaining compartments. The model assumes that symptomatic individuals will not be vaccinated, hence no vaccination arrow exiting the I_u0 compartment.
