# Supplementary material for: Informing policy via dynamic models: Cholera in Haiti
Source: PLoS Comput Biol. 2024 Apr 29;20(4):e1012032. doi: 10.1371/journal.pcbi.1012032 (PMC11081515; doi:10.1371/journal.pcbi.1012032)
Supplement: S1 Table — Conversions between the notation used here and the notation of Lee et al. [4]. (PDF) [file pcbi.1012032.s004.pdf]

Parameter	Our Notation	Lee et al. (2020a)		
		1	2	3
Reporting Rate	ρ	ρ	ρ	ϵ_1, ϵ_2
Mixing Coefficient	ν	ν	—	—
Measurement Over-Dispersion	ψ	τ	—	p
Birth Rate	μ_S	μ	—	—
Natural Mortality Rate	δ	δ	—	μ
Cholera Mortality Rate	δ_C	—	—	α
Latent Period	$1/\mu_{EI}$	$1/\sigma$	$1/\gamma_E$	—
Recovery Rate	μ_{IR}	γ	γ	γ
Loss of Immunity	μ_{RS}	α	σ	ρ
Symptomatic Ratio	f	$1-\theta_0$	k	σ
Asymptomatic Relative Infectiousness	ϵ	$1-\kappa$	red_β	—
Human-to-Water Shedding	μ_W	—	μ	θ_I
Asymptomatic Relative Shedding	ϵ_W	—	red_μ	θ_A/θ_I
Seasonal Amplitude	a	—	α_s	λ
Transmission	β	β	β	c
Water-to-Human	β_W	—	β_W	β
Bacteria Mortality	δ_W	—	δ	μ_β
Vaccination Efficacy	θ	θ_{vk}	$\theta_1, \theta_2, \theta_{1_5}, \theta_{2_5}$	η_{1d}, η_{2d}
Process Over-dispersion	σ_{proc}	—	—	σ_w
