# Supplementary material for: Informing policy via dynamic models: Cholera in Haiti
Source: PLoS Comput Biol. 2024 Apr 29;20(4):e1012032. doi: 10.1371/journal.pcbi.1012032 (PMC11081515; doi:10.1371/journal.pcbi.1012032)
Supplement: S1 Text — In depth description of the Markov chain and differential equation interpretations of compartment flow rates, as well as details on the numeric implementation of these models. (PDF) [file pcbi.1012032.s005.pdf]

Markov chain and differential equation interpretations of compartment flow rates

In the Materials and methods Section of the main article, we define compartment models in terms of their flow rates. For a discrete population model, these rates define a Markov chain. For a continuous and deterministic model, the rates define a system of ordinary differential equations. Here, we add additional details to clarify the mapping from a collection of rate functions to a fully specified process. Our treatment follows [1].

A general compartment model is a vector-valued process $X(t) = (X_1(t), \dots, X_c(t))$ denoting the (integer or real-valued) counts in each of c compartments, where t is any continuous value in the interval $[t_0, \infty)$ for some real valued starting time t_0. The compartments may also have names, but to set up general notation we simply refer to them by their numerical index. The basic characteristic of a compartment model is that $X(t)$ can be written in terms of the flows $N_{ij}(t)$ from i to j. A flow into compartment i from outside the system is denoted by $N_{\bullet i}$, and a flow out of the system from compartment i is denoted by $N_{i\bullet}$. We call $\bullet$ a source/sink compartment, though it is an irregular compartment since $X_\bullet(t)$ is not defined. These flows are required to satisfy a "conservation of mass" identity:

$$X_i(t) = X_i(t_0) + N_{\bullet i}(t) - N_{i\bullet}(t) + \sum_{j \neq i} N_{ji}(t) - \sum_{j \neq i} N_{ij}(t). \tag{S1}$$

Each *flow* $N_{ij}(t)$ is associated with a *rate* function $\mu_{ij} = \mu_{ij}(t, X(t))$, where we include the possibility that i or j takes value $\bullet$.

There are different ways to use a collection of rate functions to build a fully specified model. We proceed to describe the ones we use in this paper: via a system of ordinary differential equations (Sec. S1), a simple Markov counting system (Sec. S2), and an over-dispersed Markov counting system (Sec. S3). Other representations include stochastic differential equations driven by Gaussian noise or Gamma noise [2].

S1 Ordinary differential equation (ODE) interpretation

A basic deterministic specification is

$$dN_{ij}/dt = \mu_{ij}\big(t, X(t)\big) X_i(t), \quad i \in 1\!:\!c, \quad j \in 1\!:\!c \cup \{\bullet\}, \quad i \neq j, \tag{S2}$$

where $\mu_{ij}\big(t, X(t)\big)$ is called a per-capita rate or a unit rate. Flows into the system require special treatment since $X_i(t)$ in (S2) is not defined for $i = \bullet$. Instead, we specify

$$dN_{\bullet i}/dt = \mu_{\bullet i}\big(t, X(t)\big). \tag{S3}$$

This is the the interpretation and implementation used for Model 2 in our study.

S2 Simple Markov counting system interpretation

A continuous time Markov chain can be specified via its infinitesimal transition probabilities. A basic approach to this is to define

$$\mathbb{P}\big[N_{ij}(t+\delta) - N_{ij}(t) = 0 \,|\, X(t)\big] = 1 - \delta\mu_{ij}\big(t, X(t)\big) X_i(t) + o(\delta), \tag{S4}$$

$$\mathbb{P}\big[N_{ij}(t+\delta) - N_{ij}(t) = 1 \,|\, X(t)\big] = \delta\mu_{ij}\big(t, X(t)\big) X_i(t) + o(\delta), \tag{S5}$$

for $i \in 1\!:\!c$ and $j \in 1\!:\!c \cup \{\bullet\}$ with $i \neq j$. As with the ODE case, we need special attention for flows into the system, and we define

$$\mathbb{P}\big[N_{\bullet i}(t+\delta) - N_{\bullet i}(t) = 0 \,|\, X(t)\big] \;=\; 1 - \delta\mu_{\bullet i}\big(t, X(t)\big) + o(\delta), \tag{S6}$$

$$\mathbb{P}\big[N_{\bullet i}(t+\delta) - N_{\bullet i}(t) = 1 \,|\, X(t)\big] \;=\; \delta\mu_{\bullet i}\big(t, X(t)\big) + o(\delta). \tag{S7}$$

Together with the initial conditions $X(0)$, equations (S4)–(S7) define a Markov chain. Each flow is a simple counting process, meaning a non-decreasing integer-valued process that only has jumps of size one. We therefore call the Markov chain a simple Markov counting system (SMCS). The infinitesimal mean of every flow is equal to its infinitesimal variance [3] and so an SMCS is called equidispersed. We note that the special case of Model 1 used by [4] (with $\sigma_{\text{proc}} = 0$) is an SMCS. To permit more general mean-variance relationships for a Markov counting system, we must permit jumps of size greater than one. The utility of over-dispersed models, where the infinitesimal variance of the flow exceeds the infinitesimal mean, has become widely recognized [5, 6].

S3 Overdispersed Markov counting system interpretation

Including white noise in the rate function enables the possibility of an over-dispersed Markov counting system [1, 3, 6]. Since rates should be non-negative, Gaussian noise is not appropriate and gamma noise is a convenient option that has found various applications [7, 8]. Specifically, we consider a model given by

$$\mu_{ij}\big(t, X(t)\big) = \bar{\mu}_{ij}\big(t, X(t)\big)\, d\Gamma_{ij}(t)/dt, \tag{S8}$$

where $\Gamma_{ij}(t)$ is a stochastic process having independent gamma distributed increments, with

$$\mathbb{E}\big[\Gamma_{ij}(t)\big] = t, \quad \text{Var}\big[\Gamma_{ij}(t)\big] = \sigma_{ij}^2 t. \tag{S9}$$

Formally interpreting the meaning of (S8) is not trivial, and we do so by constructing a Markov process $X(t)$ as the limit of the Euler scheme described in Section S2, below. Therefore, the numerical scheme in Sec. S2 can be taken as a definition of the meaning of (S8). The Markov chain defined by the limit of this Euler scheme as the step size decreases is an over-dispersed Markov counting system, with the possibility of instantaneous jumps of size greater than one [3].

Numerical solutions to compartment models

Models may be fitted and their implications assessed via numerical solutions (i.e., simulations) from the model equations. All the analyses we consider have this simulation-based property, known as plug-and-play or equation-free or likelihood-free. The numerical solutions to the model are arguably of more direct scientific interest than the exact solutions to the postulated equations. For ODE models, numerical methods are well studied and a standard numerical solution package such as `deSolve` in `R` is adequate for many purposes. For SMCS and ODMCS models, exact schemes are feasible when the number of events is small, which may be the case for small populations. However, for applicability to larger populations, we use instead the following Euler scheme. Write δ for an Euler time step, and ΔN_{ij} for the numerical approximation to $N_{ij}(t+\delta) - N_{ij}(t)$ given $X(t)$. For each i and j in $1 : c \cup \{\bullet\}$ with $i \neq j$, we draw independent Gamma distributed noise increments

with mean δ and variance $\sigma_{ij}^2\delta$, denoted using a mean-variance parameterization of the gamma distribution as

$$\Delta\Gamma_{ij} \sim \text{gamma}(\delta, \sigma_{ij}^2\delta). \tag{S10}$$

In the case of an SMCS model, $\sigma_{ij} = 0$ for all i and j, so we have $\Delta\Gamma_{ij} = \delta$. Then, for $i \neq \bullet$ and $j \neq i$, and writing

$$\mu_{ij} = \bar{\mu}_{ij}\big(t, X(t)\big)\Delta\Gamma_{ij}/\delta, \tag{S11}$$

we calculate transition probabilities

$$p_{ij} = \exp\left\{-\sum_{k\in 1:c\,\cup\{\bullet\}} \mu_{ik}\,\delta\right\} \frac{\mu_{ij}}{\sum_{k\in 1:c\cup\{\bullet\}} \mu_{ik}}, \tag{S12}$$

$$p_{ii} = 1 - \sum_{j\neq i} p_{ij}. \tag{S13}$$

These probabilities correspond to competing hazards for every individual in compartment i to transition to some compartment j, interpreting $j = i$ to mean that the individual remains in i. Then, $\big(\Delta N_{i1}, \dots, \Delta N_{ic}, \Delta N_{i\bullet}\big)$ has the multinomial distribution where $X_i(t)$ individuals are allocated independently to $1 : c \cup \{\bullet\}$ with probabilities given by (S12) and (S13). We use the `reulermultinom` function in the `pomp` package to draw from this multinomial distribution.

Different treatments of demographic flows—such as birth, death, immigration and emigration—are possible. For the case $i = \bullet$, the treatment used by Model 1 is to set

$$\Delta N_{\bullet j} \sim \text{poisson}(\mu_{\bullet j}\delta), \tag{S14}$$

an independent Poisson random variable with mean $\mu_{\bullet j}\delta$.

Models 2 and 3 used an alternative approach, balancing the total number of flows in and out of the compartment, i.e., $\sum_i N_{\bullet i}(t) = \sum_i N_{i\bullet}(t)$, in order to make the model consistent with the known total population. In this case, we formally model the death rate as a rate of returning to the susceptible class S, and use external transitions from $\bullet$ into S to describe only net population increase.

References

[1] Bretó C, He D, Ionides EL, King AA. Time Series Analysis via Mechanistic Models. Annals of Applied Statistics. 2009;3:319–348.

[2] Bhadra A, Ionides EL, Laneri K, Pascual M, Bouma M, Dhiman RC. Malaria in Northwest India: Data Analysis via Partially Observed Stochastic Differential Equation Models Driven by Lévy Noise. Journal of the American Statistical Association. 2011;106:440–451.

[3] Bretó C, Ionides EL. Compound Markov Counting Processes and their Applications to Modeling Infinitesimally Over-Dispersed Systems. Stochastic Processes and their Applications. 2011;121:2571–2591.

[4] Lee EC, Chao DL, Lemaitre JC, Matrajt L, Pasetto D, Perez-Saez J, et al. Achieving Coordinated National Immunity and Cholera Elimination in Haiti Through Vaccination: A Modelling Study. The Lancet Global Health. 2020;8(8):e1081–e1089.

[5] Stocks T, Britton T, Höhle M. Model Selection and Parameter Estimation for Dynamic Epidemic Models via Iterated Filtering: Application to Rotavirus in Germany. Biostatistics. 2020;21(3):400–416.

[6] He D, Ionides EL, King AA. Plug-and-Play Inference for Disease Dynamics: Measles in Large and Small Towns as a Case Study. Journal of the Royal Society Interface. 2010;7:271–283.

[7] Romero-Severson E, Volz E, Koopman J, Leitner T, Ionides E. Dynamic Variation in Sexual Contact Rates in a Cohort of HIV-Negative Gay Men. American Journal of Epidemiology. 2015;182:255–262. doi:10.1093/aje/kwv044.

[8] Subramanian R, Romeo-Aznar V, Ionides E, Codeço CT, Pascual M. Predicting Re-Emergence Times of Dengue Epidemics at Low Reproductive Numbers: DENV1 in Rio de Janeiro, 1986–1990. Journal of the Royal Society Interface. 2020;17(167):20200273.
