# Supplementary material for: Informing policy via dynamic models: Cholera in Haiti
Source: PLoS Comput Biol. 2024 Apr 29;20(4):e1012032. doi: 10.1371/journal.pcbi.1012032 (PMC11081515; doi:10.1371/journal.pcbi.1012032)
Supplement: S2 Text — Additional details of the initialization model that were used for Models 1–3. (PDF) [file pcbi.1012032.s006.pdf]

S1 Initial Values

To perform inference on POMP models, it is necessary to propose an initial probability density for the latent process $f_{X_0}(x_0; \theta)$, including the possibility that the initial values of the latent states are known, or are a non-random function of the unknown parameter vector, θ. This density is used to obtain initial values of the latent state when fitting and evaluating the model. For each of the models considered in this analysis, the initial conditions are derived by enforcing the model dynamics on reported cholera cases. It is also sometimes necessary to fit some initial value parameters in order to help determine initial values for weakly identifiable compartments. In the following subsections, we mention initial value parameters that were fit for each model.

S1.1 Model 1

For this model, the number of individuals in the Recovered and Asymptomatic compartments are set to zero, but the initial proportion of Infected and Exposed individuals is estimated as initial value parameters ($I_{0,0}$ and $E_{0,0}$, respectively) using the IF2 algorithm, implemented as `mif2` in the `pomp` package. Finally, the initial proportion of Susceptible individuals $S_{0,0}$ is calculated as $S_{0,0} = 1 - I_{0,0} - E_{0,0}$. This model for the initial values of the latent states matches that which was used by Lee et al. (2020) [1].

S1.2 Model 2

Model 2 assumes that the initial values are a deterministic function of the reporting rate and the initial case reports, and so no initial value parameters need to be estimated. Initial values for latent state compartments are chosen so as to enforce the model dynamics on the observed number of cases. Specifically, the model sets $I_{u0}(0) = y^*_{1u}/\rho$ for each unit $u \in 1:10$, where $I_{u0}(t)$ is the number of infected individuals in unit u at time t, vaccination scenario $z = 0$, y^*_{tu} is the reported number of cases, and ρ is the reporting rate. It is further assumed that there are no individuals in the recovered compartment, as the epidemic has just begun. This model for the initial values of the latent states matches that which was used by Lee et al. (2020) [1].

The decision to fix initial values so that they satisfy the dynamics of the model has the benefit of reducing the number of estimated parameters and enforcing latent states at time t_0 to be consistent with the calibrated model. The risk of fixing initial values rather than estimating them is doing so may have substantial effects on the model dynamics, and hence on the consequences of the analysis. To consider the impact of the chosen model approach, we consider an alternative initialization model that enables flexible estimation of certain latent states. Specifically, we initialize $I_{u0}(0) = \tilde{I}_{u0}$ for $u \in 1:10$, where $\{\tilde{I}_{u0}\}_{u=1}^{10}$ is a set of additional model parameters. We then fix $S_{u0}(0) = \text{pop}_u - I_{u0}(0)$, and all other starting values are set to zero, as with the fixed value approach.

The AIC of this alternative approach is 43854.0, compared to the fixed approach with an AIC of 43926.5. This alternative initialization approach results in quantitative improvement to the model-fit, but does not result in qualitative differences in the conclusions made using this model. Figure S-1 displays the trajectory of the model with this alternate initialization model. Table S-1 gives the estimated initial values. The estimated value of the latent state is similar to the fixed value in Artibonite and Centre, where the largest number of cases are present at the start of the epidemic. Because of this, the qualitative dynamics do not differ by much when the parameters are estimated versus held constant, despite the improvement in model fit measured by AIC.

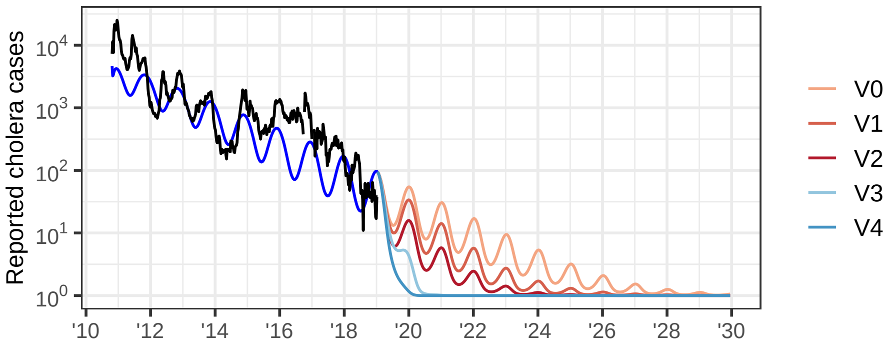

Fig S-1: **Simulated trajectory of alternate initialization of Model 2.** The black line shows the nationally aggregated weekly cholera incidence data. The blue curve from 2012-2019 is the trajectory of the calibrated version of Model 2. Compare to Fig. 4 of the article.

Table S-1: Initial values estimated for alternative initialization model for Model 2 compared to the fixed-value initialization model.

Latent State	Department	Calibrated Model Value	Fixed Value
$I_{1,0}(0)$	Artibonite	27125	31170
$I_{2,0}(0)$	Centre	2095	2535
$I_{3,0}(0)$	Grand'Anse	64	0
$I_{4,0}(0)$	Nippes	35	0
$I_{5,0}(0)$	Nord	0	85
$I_{6,0}(0)$	Nord-Est	0	0
$I_{7,0}(0)$	Nord-Ouest	3233	10
$I_{8,0}(0)$	Ouest	878	2700
$I_{9,0}(0)$	Sud	50	0
$I_{10,0}(0)$	Sud-Est	0	0

S1.3 Model 3

The latent states of this model are initialized by enforcing the model dynamics on the incidence data from the start of the recorded cases until time t_0, requiring that some of the available data be used to determine the initial values of the latent states. This is the same approach that was taken by Lee et al. (2020) [1], who used the value t_0 = 2014-02-22; this choice of t_0 results in modeling roughly only 60% of the available data, some of which is later discarded for alternative reasons [2].

We do not see any immediate reason that this model could not be extended to cover a larger range of the data, and chose the value t_0 = 2010-11-13. This choice of t_0 corresponds to using approximately 1% of the available data to determine initial values of the latent states. In addition to modeling a larger portion of the available data, this choice of t_0 corresponds to an important real-world event, as daily reports from each of the departments were not being sent to the health ministry until November 10, 2010 [3]; this choice of t_0 therefore makes $\boldsymbol{Y}(t_1)$ the first week of data once daily reports are being sent to the health ministry. The few observation times that exist before t_0 are used to initialize the model by enforcing model dynamics on these preliminary observations.

For convenience, we denote these observations as t_{-3}, t_{-2} and t_{-1}; as before, we let $y_{u,-k} = Y_u(t_{-k})$ denote the observed case count for unit u at time point t_{-k}, where $k \in 1:3$. Equations for the initial values of non-zero latent states are provided in Eqs. (S1)–(S5); these equations match those that were used by Lee et al. (2020) [1], the primary change being a change to the value of t_0.

$$
\begin{aligned}
I_u(t_0) &= \frac{y^*_{u,-1}}{7\rho\left(\mu_{IR} + (\delta + \delta_C)/365\right)}, && \text{(S1)} \\
A_u(t_0) &= \frac{I_{u0}(t_0)\,(1-f)}{f}, && \text{(S2)} \\
R_{u01}(t_0) &= R_{u02}(t_0) = R_{u03}(t_0) = \left(\frac{\sum_{k=-3}^{0} y^*_{u,k}}{\rho f} - \left(I_{u0}(t_0) + A_{u0}(t_0)\right)\right)/3 && \text{(S3)} \\
S_{u0}(t_0) &= \text{Pop}_u - I_u(t_0) - A_u(t_0) - \sum_{k=1}^{3} R_{u0k}(t_0) && \text{(S4)} \\
B_u(t_0) &= \left[1 + a\tilde{J}^r\right]\text{Den}_u\,\mu_W\left[I_u(t_0) + \epsilon_W A_{u_0}(t)\right]/\mu_W. && \text{(S5)}
\end{aligned}
$$

In Eq. (S5), $\tilde{J} = 0.002376$ is the median adjusted rainfall over the observation period. One important consideration to make with this parameter initialization model is when $y^*_{u,-1} = 0$, which occurs for units $u \in \{3,4\}$, which correspond to the Grand'Anse and Nippes departments, respectively. When this is the case, each of the infectious $I_u(t_0)$, asymptomatic $A_u(t_0)$, and bacterial reservoir $W_u(t_0)$ compartments have a value of zero. Because Model 3 models cholera transmission primarily by means of the bacterial reservoir, this makes it nearly impossible for an outbreak to occur. Therefore for units $u \in \{3,4\}$, we introduce initial value parameters $I^3_{0,0}$ and $I^4_{0,0}$, and calibrate these parameter values using the data. The resulting parameter estimates are used to obtain the remaining non-zero initial values of the latent states using Eqs. (S2)–(S5).

References

[1] Lee EC, Chao DL, Lemaitre JC, Matrajt L, Pasetto D, Perez-Saez J, et al. Achieving Coordinated National Immunity and Cholera Elimination in Haiti Through Vaccination: A Modelling Study. The Lancet Global Health. 2020;8(8):e1081–e1089.

[2] Lee EC, Chao DL, Lemaitre JC, Matrajt L, Pasetto D, Perez-Saez J, et al. Supplement to: Achieving Coordinated National Immunity and Cholera Elimination in Haiti Through Vaccination: A Modelling Study. The Lancet Global Health. 2020;8(8):e1081–e1089.

[3] Barzilay EJ, Schaad N, Magloire R, Mung KS, Boncy J, Dahourou GA, et al. Cholera Surveillance During the Haiti Epidemic—the First 2 Years. New England Journal of Medicine. 2013;368(7):599–609.
