# Supplementary material for: Informing policy via dynamic models: Cholera in Haiti
Source: PLoS Comput Biol. 2024 Apr 29;20(4):e1012032. doi: 10.1371/journal.pcbi.1012032 (PMC11081515; doi:10.1371/journal.pcbi.1012032)
Supplement: S3 Text — Additional details of the measurement model that were used for Models 1–3. (PDF) [file pcbi.1012032.s007.pdf]

Measurement Models

Each POMP requires specification of a measurement model, which is a statistical description of how observations on the system are obtained. In general, we used the same measurement models that were reported by [1] unless specifically noted in the following subsections.

S1 Model 1

In this model, the advantage afforded by vaccination is an increased probability that an infection is asymptomatic. Therefore, under the assumptions of this model, all reported cases are assumed to be a fraction of individuals that transition from the exposed to the infected compartment, as noted in Eq. (S1):

$$Y_n \mid \Delta N_{E.I.}(n) = \delta \sim \mathrm{NB}\left(\rho\delta, \psi\right), \tag{S1}$$

where Y_n is the reported cholera cases at time $t_n \in t_1 : t_N$ and $\Delta N_{E.I.}(n)$ is the sum total of individuals across each vaccination compartment $z \in 1 : Z$ who moved from compartment E_z to I_z since observation t_{n-1}. Here, $\mathrm{NB}\left(\rho\delta, \psi\right)$ denotes a negative binomial distribution with mean $\rho\delta$ and variance $\rho\delta\big(1 + \frac{\rho\delta}{\psi}\big)$.

S2 Model 2

Model 2 was fit using reported case counts that were transformed using the natural logarithm. We fit Model 2 using the subplex algorithm in the `subplex` package, using a Gaussian measurement model (Eq. (S2)) on the log transformed cases within each unit. The final loss function that is maximized is the product of the likelihoods of the individual units, or the sum of the log-likelihood of the individual units. The measurement model for individual units is given in Eq. (S2).

$$\log\big(Y_{u,n} + 1\big) \mid \Delta N_{E_u \cdot I_u \cdot}(n) = \delta_u \sim \mathrm{N}\left(\log\big(\rho\delta_u + 1\big), \psi^2\right), \tag{S2}$$

where $\Delta N_{E_u \cdot I_u \cdot}(n)$ is the sum total of individuals across vaccination compartment $z \in 0 : 4$ within unit u who moved from compartment E_{uz} to I_{uz} since observation t_{n-1}. Therefore, because the natural logarithm of observed case counts (plus one, to avoid taking the logarithm of zero) has a normal distribution, $Y_{u,n}+1$ is assumed to follow a log-normal distribution with log-mean parameter $\log\big(\rho\Delta N_{E_u \cdot I_u \cdot}(n) + 1\big)$ and log-variance ψ^2. We note that fitting a model with this measurement model is equivalent to fitting using least squares, with $\log(Y_{u,n} + 1)$ as the response variable.

This measurement model differs from that used by Lee et al. (2020) [1], who fit the model in two stages: epidemic and endemic phases. Although their text and supplement material do not explicitly describe the measurement model used, inspection of the code provided with their submission suggests a change in measurement model between the epidemic and endemic phases. The measurement model they used for the epidemic phase is

$$Y_{u,n} \mid \Delta N_{E_u \cdot I_u \cdot}(n) = \delta_u \sim \mathrm{N}\left(\rho\delta_u, \psi^2\right), \tag{S3}$$

The measurement model they used for the endemic phase modifies the epidemic model by counting both asymptomatically infected (A) and symptomatically infected (I) individuals in the case counts:

$$Y_{u,n} \mid \Delta N_{E_u \cdot I_u \cdot}(n) = \delta_{u1}, \Delta N_{E_u \cdot A_u \cdot}(n) = \delta_{u2} \sim \mathrm{N}\left(\rho\big(\delta_{u1} + \delta_{u2}\big), \psi^2\right), \tag{S4}$$

where the notation for $\Delta N_{E_u \cdot A_u \cdot}(n)$ is similar to $\Delta N_{E_u \cdot I_u \cdot}(n)$, described above.

S3 Model 3

In this model, reported cholera cases are assumed to stem from individuals who develop symptoms and seek healthcare. Therefore reported cases are assumed to come from an over-dispersed negative binomial model, given the increase in infected individuals:

$$Y_{u,n} \mid \Delta N_{S_u \cdot I_u}(t) = \delta_u \sim \mathrm{NB}\left(\rho\delta_u, \psi\right), \tag{S5}$$

where $\Delta N_{S_u \cdot I_{uz}}(n)$ is the number of individuals who moved from compartment S_{uz} to I_u since observation t_{n-1}.

This measurement model is a minor change from that used by [1], which allowed for a change in the reporting rate on January 1st, 2018. The fitted values of the reporting rate—before and after January 2018—were 0.97 and 0.097, respectively. An instantaneous change from near perfect to almost non-existent reporting can be problematic, as it forces the model to explain the observed reduction in reported cases as a decrease in the reporting of cases, rather than a decrease in the prevalence of cholera. This shift was justified by a "change of the case definition that occurred on January 1st, 2018"; this claim was not cited, and we could find no evidence that such a drastic change in the reporting rate would be warranted. We therefore do not allow a change in reporting rate when fitting Model 3.

References

[1] Lee EC, Chao DL, Lemaitre JC, Matrajt L, Pasetto D, Perez-Saez J, et al. Achieving Coordinated National Immunity and Cholera Elimination in Haiti Through Vaccination: A Modelling Study. The Lancet Global Health. 2020;8(8):e1081–e1089.
