# Supplementary material for: Informing policy via dynamic models: Cholera in Haiti
Source: PLoS Comput Biol. 2024 Apr 29;20(4):e1012032. doi: 10.1371/journal.pcbi.1012032 (PMC11081515; doi:10.1371/journal.pcbi.1012032)
Supplement: S4 Text — Details of how the log-likelihood of the Lee et al. [4] models were calculated. (PDF) [file pcbi.1012032.s008.pdf]

Replication of Lee et al. (2020) [1]

In this article we claimed that we were able to obtain better fits to the observed data using the same models that were proposed by Lee et al. (2020) [1]. Along with visual comparisons to the data, this claim was supported by comparing likelihoods and AIC values in Table 2 in the manuscript. Because model likelihoods were not provided by Lee et al. (2020) [1], it is necessary to replicate these models in order to obtain likelihood estimates. Here we would like to thank the authors of Lee et al. (2020) [1], who provided detailed descriptions of their models, which enabled us to build on their work. In the following subsections, we use our `R` package `haitipkg` to reproduce some of the results of [1]. This reproduction allows us to estimate the likelihoods of the Lee et al. (2020) [1] version of Models 1–3, and also provides a demonstration of the importance and usefulness of reproducible research.

S1 Model 1 Replication

The model was implemented by a team at Johns Hopkins Bloomberg School of Public Health (hereafter referred to as the Model 1 authors) in the `R` programming language using the `pomp` package [2]. Original source code is publicly available with DOI: 10.5281/zenodo.3360991. The final results reported by the Model 1 authors were obtained by using several different parameter sets rather than a single point estimate. According to the supplement materials, this was because model realizations from a single parameter set retained substantial variability, but multiple realizations from a collection of parameter sets resulted in a reasonable visual fit to the data. We are also inclined to believe that the use of multiple parameter values was in part intended to account for parameter uncertainty—the importance of which was discussed in the main text—an effort by the Model 1 authors that we applaud. Simulations from each of the parameter sets, however, were treated with equal importance when being used to diagnose the model fit and make inference on the system. This is problematic given Figures S8 and S9 of the supplement material, which suggest that some parameter sets that were used for inference may have been several hundred units of log-likelihood lower than other parameter sets that were simultaneously used to make forecasts. Such a large difference in log-likelihoods is well beyond the threshold of statistical uncertainty determined by Wilks' theorem, resulting in the equal use of statistically inferior parameter sets in order to make forecasts and conduct inference on the system.

To fully reproduce the results of the Model 1 authors, it is necessary to use the exact same set of model parameters that were originally used to obtain the results presented by [1]. Because these parameter sets were not made publicly available, we relied on the source code provided by the Model 1 authors to approximately recreate the parameter set. Due to software updates since the publication of the source code, we were unable to produce the exact same set of parameters. Running the publicly available source code, however, resulted in a set of parameters that are visually similar to those used by the Model 1 authors (See Figures S-1 and S-2). Furthermore, simulations using the set of parameters produced by the source code appear practically equivalent to those displayed by [1] (See Figure S-3).

Because the model forecasts provided by [1] come from various sets of parameters—which each correspond to a unique log-likelihood value—it is not obvious how one would obtain an estimate for the log-likelihood of the model that was used for simulations by the Model 1 authors. One approach could be to calculate the logarithm of the weighted mean of the likelihoods for each parameter sets used to obtain the forecasts, where the weights are proportional to the number of

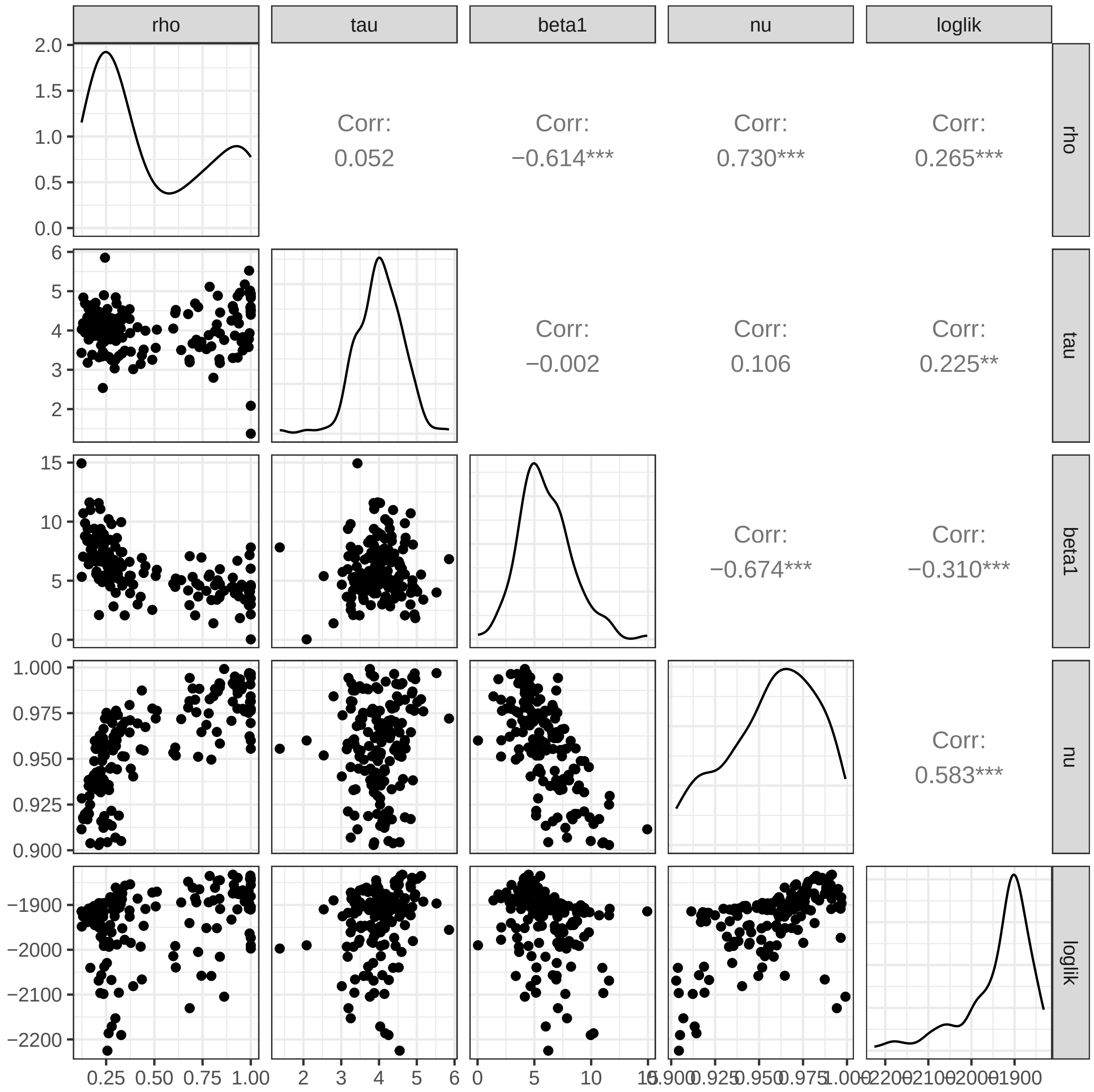

Fig S-1: Bivariate distributions of parameter estimates after fitting epidemic phase of the Model 1 following the procedure described by Lee et al. (2020) [1]. Compare to Figure S8 in the supplement of Lee et al. (2020) [1].

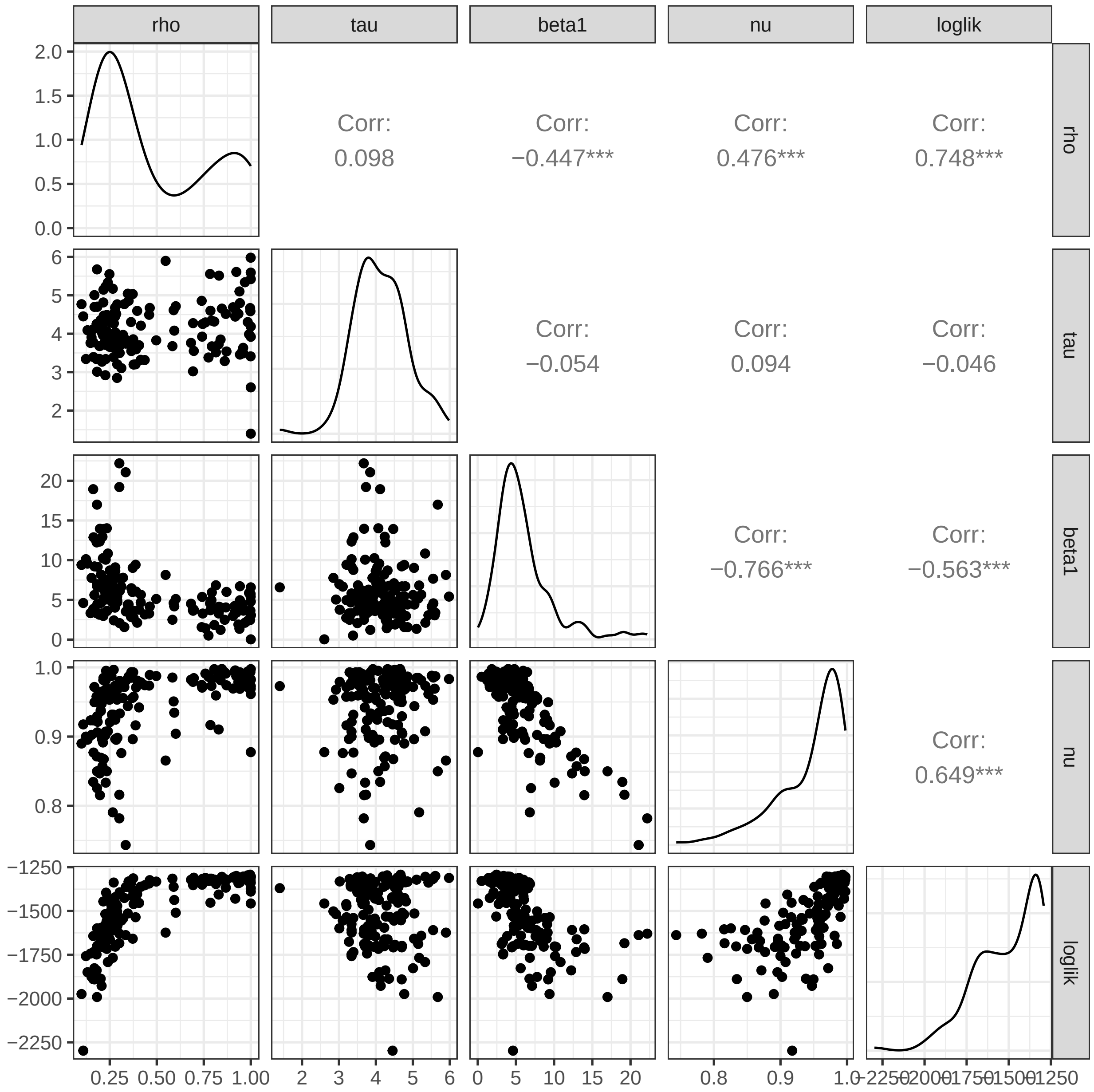

Fig S-2: Bivariate distributions of parameter estimates after fitting endemic phase of the Model 1 following the procedure described by Lee et al. (2020) [1]. Compare to Figure S9 in the supplement of Lee et al. (2020) [1].

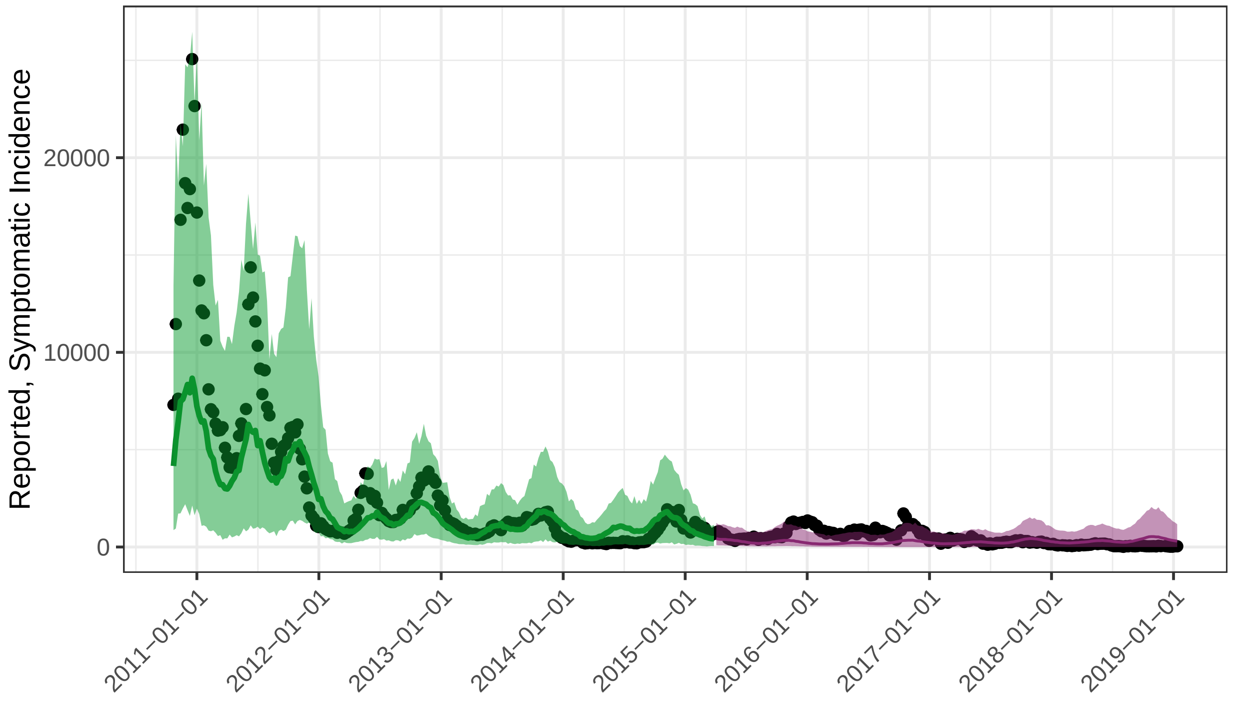

Fig S-3: Simulations from Model 1 using parameter sets that were generated by running source code provided by Lee et al (2020) [1]. Compare to Figure S7 in the supplement of Lee et al. (2020) [1]. The upper bound for the likelihood of this model is -3031.

times the parameter set was used. However, in an effort to not underestimate the likelihood of the model of the Model 1 authors, we report the estimated log-likelihood as the log-likelihood value corresponding to the parameter set with the largest likelihood value, even though the majority of simulations were obtained using parameter sets with lower likelihood values. In this sense, we consider the log-likelihood reported in Table 1 of the main text to be an upper-bound of the log-likelihood of the model used by [1]. For each parameter set, the log-likelihood was estimated using a particle filter, implemented as the `pfilter` function in the `pomp` package.

S2 Model 2 Replication

Model 2 was developed by a team that consisted of members from the Fred Hutchinson Cancer Research Center and the University of Florida (hereafter referred to as the Model 2 authors). While Model 2 is the only deterministic model we considered in our analysis, it contains perhaps the most complex descriptions of cholera in Haiti: Model 2 accounts for movement between spatial units; human-to-human and environment-to-human cholera infections; and transfer of water between spatial units based on elevation charts and river flows.

The source code that the Model 2 authors used to generate their results was written in the `Python` programming language, and is publicly available at `10.5281/zenodo.3360857` and its accompanying GitHub repository `https://github.com/lulelita/HaitiCholeraMultiModelingProject`. In order to perform our analysis in a unified framework, we re-implemented this model in the `R` programming language using the `spatPomp` package [3], which facilitates the creation of meta-population models. We note that the travel and water matrices used to implement the complex dynamics in Model 2 [1] are not available in either the Zenodo archive or the GitHub repository; instead, we obtained these matrices via personal correspondence with the Model 2 authors. Using these matrices, and the point estimates for model parameters provided by [1], we created trajectories of the cholera dynamics and compared this to available data. These trajectories, shown in Figure S-4, are very similar to the trajectories shown in Figure S15 of the supplement of [1].

There are minor differences between Figure S-4 and Figure S15 of [1]. While the discrepancy appears minor, the deterministic nature of Model 2 implies that an exact replication of model trajectories should be possible. In this case, these discrepancies may possibly be attributed to implementing the model and plotting the model trajectory in two different programming languages. Another potential explanation for the discrepancy is that the parameters that we used are only approximately the same as those used by Lee et al. (2020) [1]. For example, the parameters β, β_W had reported values of 9.9×10^{-7} and 4.03×10^{-2}, respectively (Table S13 of the supplement material of [1]), but were actually fit to data and therefore likely these values have been rounded. Additionally, our implementation of Model 2 used a time scale of years and many of the parameters were reported on a weekly scale, so small differences may result due to unit conversions. The collective effect of these small differences in model parameters likely will result in small differences in model trajectories.

Some additional concerns about being able to accurately replicate the results of [1] are valid. Details about the measurement models and how latent states were initialized for the epidemic model were not provided by Lee et al. (2020) [1] and therefore these details must be inferred by looking at the provided source code. According to repository comments, the files `fitInPieces3paramsCleanMay2019Public.py` and `fitInPiecesMuWithFracSusFixedAllInfectionsPublic.py` were used to fit the epidemic and endemic phases of the model respectively, although it is apparent that these

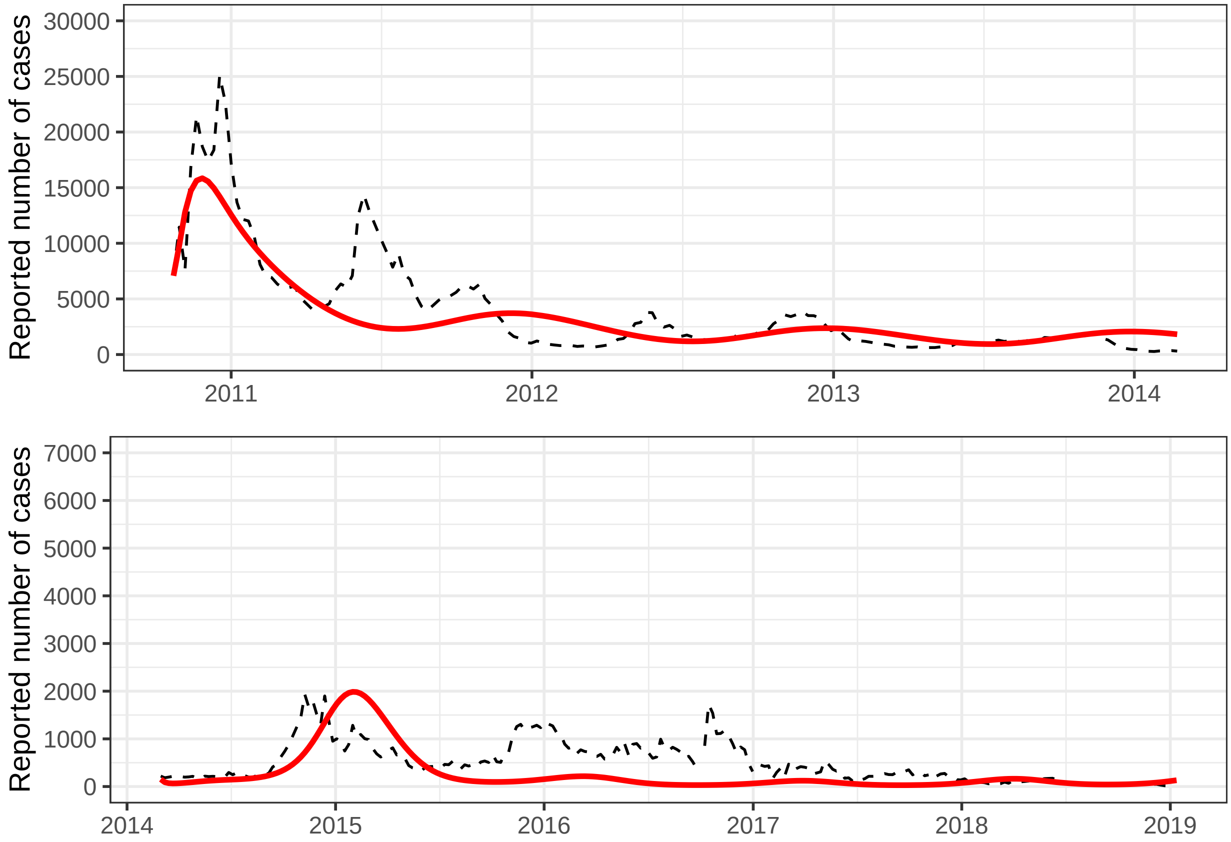

Fig S-4: Model 2 trajectories using the `haitipkg`. Compare to Figure S15 in the supplement of [1].

exact files were not used to obtain the reported results since the files contain some variable-naming errors that make it impossible to run the files without making modifications [1]. The inability to replicate the results by Lee et al. (2020) [1] by running the provided source code makes checking whether or not a our numeric implementation faithfully represents their results very difficult. Additionally, the script that was said to been used to obtain the results reported by [1] appears to use a different measurement model than what was described in the supplemental material, again making it difficult to fully replicate the result of [1] without being able to easily run the provided source code. In this case, we chose to use measurement model that considers only symptomatic individuals for both phases of the epidemic, as this seemed to visually match the results of [1] most closely.

S3 Model 3 Replication

Model 3 was developed by a team of researchers at the Laboratory of the Swiss Federal Institute of Technology in Lausanne, hereafter referred to as the Model 3 authors. The code that was originally used to implement Model 3 is archived with the DOI: `10.5281/zenodo.3360723`, and

[1] One example of why the code cannot be run that the file loads functions from a non-extant file named `choleraEqs.py` in line 13 rather than `choleraEqsPublic.py`.

also available in the public GitHub repository: `jcblemai/haiti-mass-ocv-campaign`. Because the code was made publicly available, and final model parameters were reported in the supplementary material of [1], we were able to reproduce Model 3 by directly using the source code. In Fig. S-5, we plot simulations from this model. This figure can be compared to Figure S18 of [1]. We note that slight differences may be accounted for due to variance in the model simulations and the difference in programming language used to produce the figure. Overall, the high standard of reproducibility that was achieved by the Model 3 authors facilitated the ability to readily replicate their model and results.

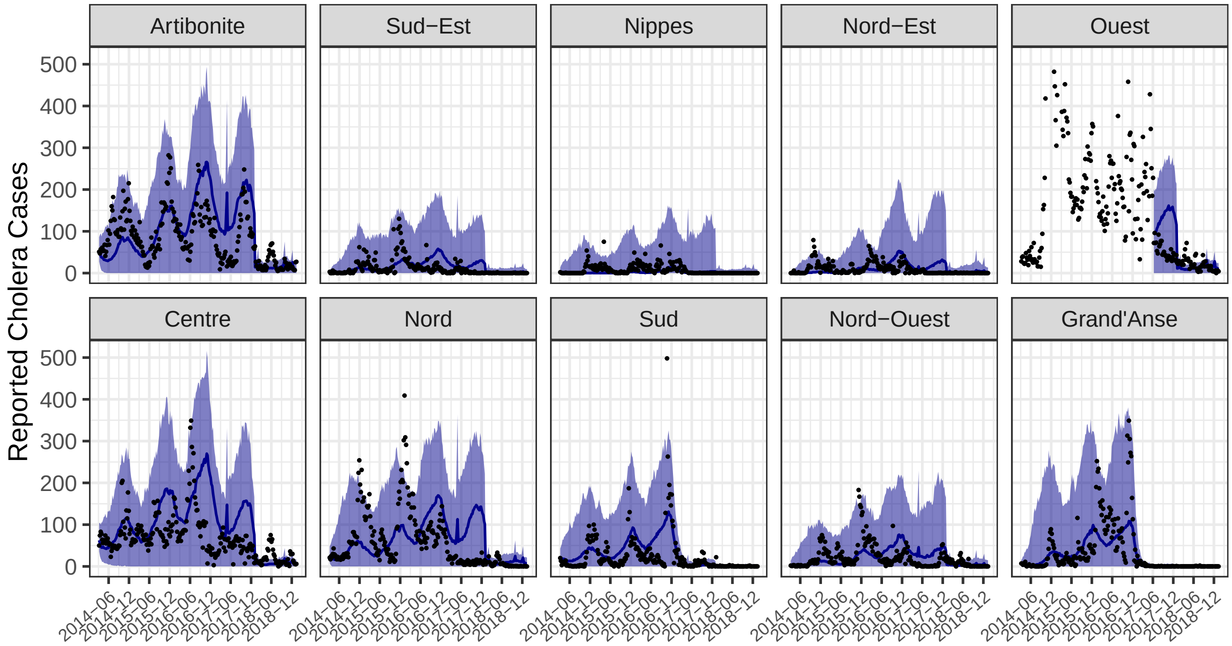

Fig S-5: Simulations from Model 3. Compare to Figure S18 in the supplement of [1].

References

[1] Lee EC, Chao DL, Lemaitre JC, Matrajt L, Pasetto D, Perez-Saez J, et al. Achieving Coordinated National Immunity and Cholera Elimination in Haiti Through Vaccination: A Modelling Study. The Lancet Global Health. 2020;8(8):e1081–e1089.

[2] King AA, Nguyen D, Ionides EL. Statistical Inference for Partially Observed Markov Processes via the R Package pomp. Journal of Statistical Software. 2016;69:1–43.

[3] Asfaw K, Park J, King AA, Ionides EL. Partially Observed Markov Processes with Spatial Structure via the R Package spatPomp. arXiv:210101157v3. 2023;.
