# Supplementary material for: Informing policy via dynamic models: Cholera in Haiti
Source: PLoS Comput Biol. 2024 Apr 29;20(4):e1012032. doi: 10.1371/journal.pcbi.1012032 (PMC11081515; doi:10.1371/journal.pcbi.1012032)
Supplement: S5 Text — Additional details on the procedures for fitting and diagnosing Model 3. (PDF) [file pcbi.1012032.s009.pdf]

Calibrating Model 3 to observed cases

In this section, we provide more detail on the process that was used to estimate the coefficients of Model 3. In particular, we discuss why we decided to include additional model parameters—those that are associated with the behavior of the system during Hurricane Matthew—that were not considered by [1]. To calibrate this model, we used the iterated block particle filter (IBPF) method of [2]. Due to the novelty of this algorithm, there exists only a few examples where the IBPF algorithm is used for data analysis [2, 3], which is one motivation of the inclusion additional details related to fitting and diagnosing the model fit provided here.

Lee et al. (2020) [1] only estimated model parameters to a simplified version of Model 3 on a subset of the available data, as no method existed at the time of their publication to fit a fully coupled meta-population model to disease incidence data. Building on their results, we fit the fully coupled version of Model 3 to (nearly) all available data, reserving only a few observations to use to calibrate the initial conditions of the model (see the supplement for initialization models for more details). To maximize model likelihoods, we relied on parameter estimates obtained while calculating profile-likelihood confidence intervals, as this calculation requires many replicated IBPF searches. In our preliminary investigations that were done prior to conducting a profile likelihood search, we found that it was necessary to use multiple searches for the MLE, periodically pruning away less successful searches. To do this, the first collection of searches is performed by obtaining initial values for the parameters by uniformly sampling values from a predefined hypercube. A subsequent refinement search used parameter values corresponding to the largest model likelihoods as starting parameter values. The need for multiple searches does not appear to be uncommon, as a similar approach was used in [2]. While computationally intensive, profile likelihoods proved to be an effective alternative to maximizing model likelihoods without the need to apply this multistage heuristic.

We use the iterative fitting / pruning technique described above to fit the fully coupled version of Model 3 proposed in [1]. The maximum likelihood we obtained after two rounds of searching was -17549, which is higher than the benchmark model (-17933). While beating a simple associative benchmark is promising, this does not immediately imply that the model is a good description of the system. Additional investigation of parameters estimates and their corresponding implications on model based conclusions should always be conducted. For meta-population models, it is worth considering how well the model fits the data to each spatial unit. The likelihoods for each department, compared to the corresponding benchmark model, are displayed in Fig. S-1. The figure demonstrates that while the log-likelihood of the fitted model outperforms the auto-regressive negative binomial benchmark model at the aggregate level, Model 3 has lower likelihoods for some departments.

In addition to considering the conditional log-likelihoods of each unit, one can consider conditional log-likelihoods of each observation. When compared to a benchmark, this level of detail can provide useful information about which observations are well described by the model and which are not. In Fig. S-2, we plot the conditional log-likelihoods of Model 3 for each observation. Typically it is most useful to compare the conditional log-likelihoods of the model under consideration to a benchmark, as plotting only conditional log-likelihoods without a comparison may not be helpful. In this case, however, the same insight can be drawn using a figure without a benchmark comparison, so we do not include the benchmark in order to avoid the issue of over-plotting.

Fig. S-2 reveals that the fitted model poorly describes certain features of the data. For example,

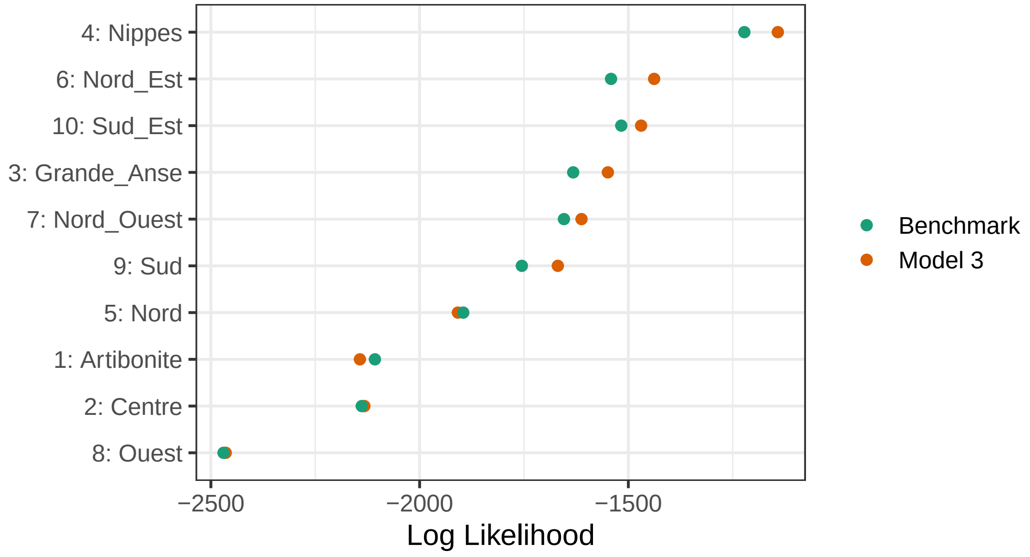

Fig S-1: Log-likelihoods of Model 3 for each department compared to the corresponding benchmark model prior to the inclusion of parameters that account for Hurricane Matthew.

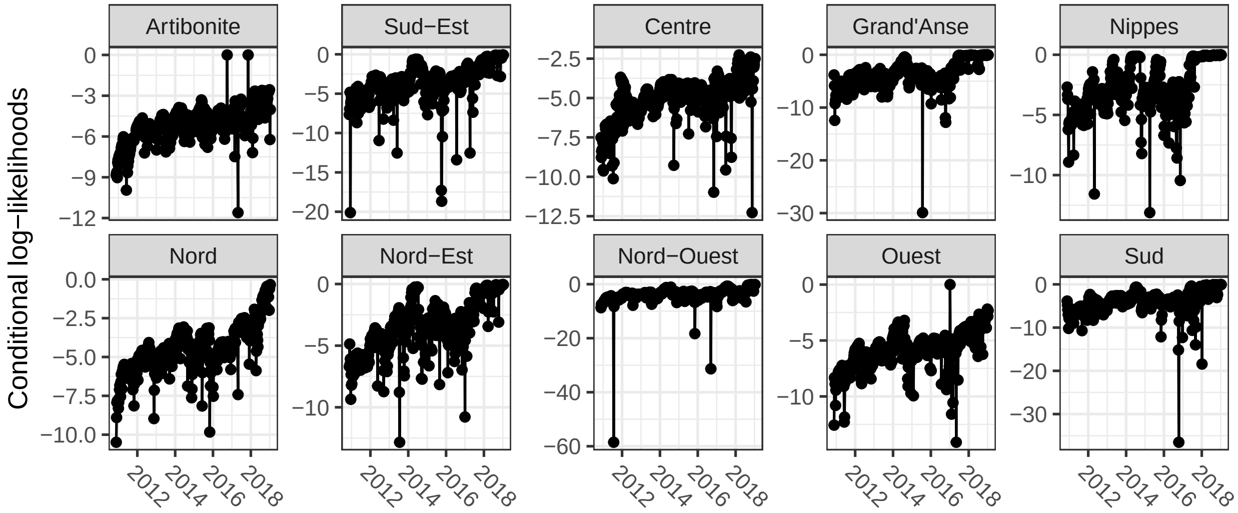

Fig S-2: Conditional log-likelihoods of Model 3 prior to the inclusion of the Hurricane Matthew related parameters.

many departments (in particular Sud) have observations with lower conditional log-likelihoods near October 2016 than at other time points. Further investigation of the model output reveals that the model is struggling to explain the sudden surge in cholera cases that occurred at this time, which coincides with the time that Hurricane Matthew struck Haiti. While the model does include a mechanism to account for increased cholera transmission due to large rainfall events, the mechanism does not appear to be sufficient to capture the damaging effects of the hurricane, which

had the greatest impact in the the Sud and Grand'Anse departments [4]. This result led us to include parameters β_{Wu}^{hm} and h_u^{hm} in Eq. 23 of the main text, which allows for an increase in the transmission rate between environmental cholera and humans for in Sud and Grand'Anse during and after the hurricane. The effect of the hurricane on cholera transmission is assumed to have an exponential decay, where the magnitude is determined by β_{Wu}^{hm} and the duration of the effect determined by h_u^{hm}.

We refit Model 3 after introducing these hurricane-related parameters. The resulting model has a log-likelihood value of -17332.9. The inclusion of these parameters resulted in an overall increase of 216.4 log-likelihood units. Such a large difference in log-likelihoods is well beyond the threshold of statistical uncertainty determined by Wilks' theorem, suggesting that the data highly favor the inclusion of the additional parameters. The addition of the Hurricane parameters also increases in conditional likelihoods for each observation, particularly around October 2016 (Fig. S-3).

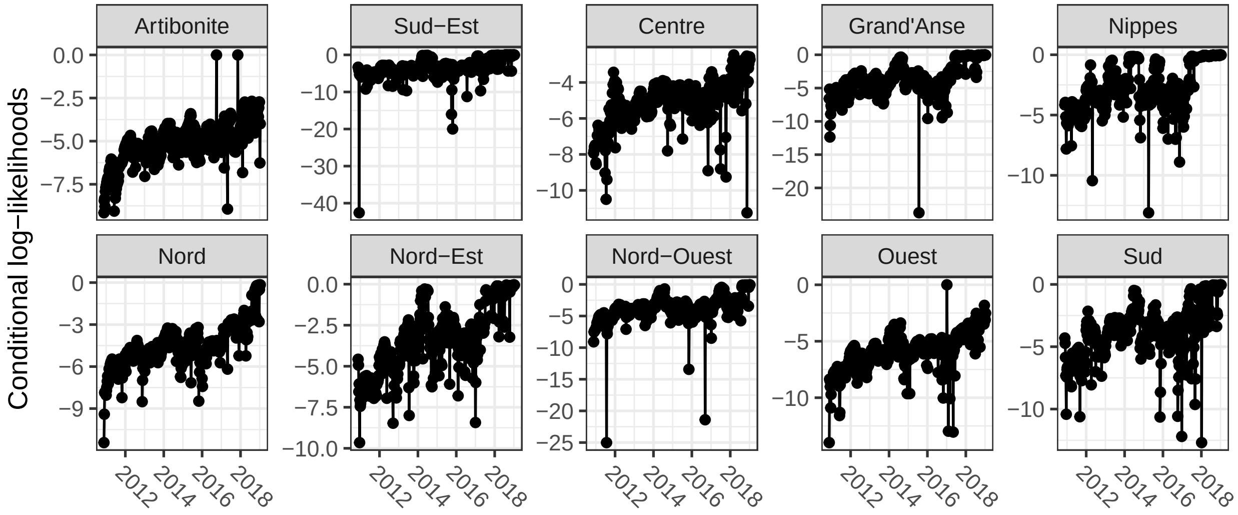

Fig S-3: Conditional log-likelihoods of Model 3 after adding and estimating the parameters related to Hurricane Matthew.

Now that the model with additional parameters has been calibrated to the incidence data, we plot the conditional log-likelihood of each department compared to a benchmark model in Fig. S-4. The difference in log-likelihoods between Model 3 and the benchmark model is smallest in the departments Artibonite, Nord, Ouest and Centre. Each of these departments also exhibited the most sustained cholera transmission, defined by having the fewest number of weeks with no recorded cholera cases. Specifically, these four departments have zero cholera cases recorded in less than 4% of the available data, and all remaining departments—except for Nord-Ouest, which has approximately 9.5% of cases that are zeros and also exhibits the next smallest difference in log-likelihoods—have zero cases recorded in at least 14% of the available weekly data. This result suggests that the quantitative advantage Model 3 has over its respective benchmark is primarily due to the model's ability to describe a resurgence of cases after a department records a week of zero cholera cases. This result may be unsurprising in the context of the models that we are comparing. Because the cholera transmission in individual departments likely depends on the national prevalence of cholera and the Vibrio cholerae bacteria, our spatially-independent benchmark model

that relies exclusively on the previous number of case within any given unit has a difficult time predicting a resurgence of cases.

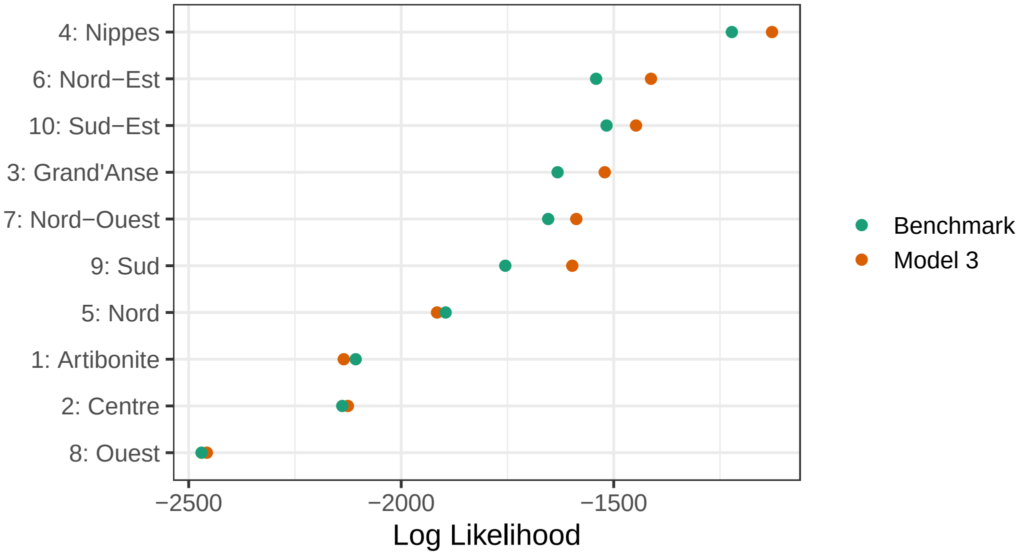

Fig S-4: Log-likelihoods of Model 3 for each department compared to the corresponding benchmark model after adding and estimating parameters related to Hurricane Matthew.

The difference in log-likelihoods between Model 3 and its benchmark model for each individual units suggests that Model 3 has a relatively poor fit for the four units with the most sustained cholera transmission. The simple four parameter benchmark has a higher likelihood than Model 3 for the Artibonite and Nord departments, and also has log-likelihoods only a few units smaller than Model 3 for the departments Ouest and Centre. This is particularly worrisome given that these four departments account for more than 77% of the total number of reported cholera cases.

S1 Examining the Hidden States of the Calibrated Model

For mechanistic models, beating a suitable statistical benchmark does not alone guarantee that the model provides an accurate description of a dynamic process. Indeed, a good statistical fit does not require the model to assert a causal explanation. For example, reconstructed latent variables should make sense in the context of alternative measurements of these variables [5]. We demonstrate this principle by examining the latent state of the calibrated model. In particular, we examine the compartment of susceptible individuals under various scenarios. This analysis can also provide insight into why the calibrated model fails to outperform the benchmark model on the four departments with the most sustained cholera transmission.

Recall that the filtering distribution for the calibrated version of Model 3 at time t_k is defined as the distribution of the latent state at time t_k given the data from times $t_1 : t_k$, i.e. $f^{(3)}_{\boldsymbol{X}_k|\boldsymbol{Y}_{1:k}}(\boldsymbol{x}_k|\boldsymbol{y}^*_{1:k};\hat{\theta})$. In general, one may expect simulations from the filtering distribution of a model with a good statistical fit to result in hidden states that are highly consistent with the observed data because the filtering distribution is conditioned on the observed data. Fig. S-5 shows the percentage of individuals that are in the susceptible compartment from various simulations

of the model: simulations from Model 3 under initial conditions are displayed in red; simulations from the filtering distribution of model are displayed in blue. This figure shows that simulations from initial conditions tends to result in a much more rapid depletion of susceptible individuals at the start of the epidemic than simulations from the filtering distribution, suggesting the calibrated model has a propensity to predict larger outbreaks than what is typically seen in the data. This result demonstrates that the calibrated model favors a more rapid growth in cholera cases than what is typically seen in the observed data, providing a possible explanation as to why the model fails to beat the simple benchmark for each spatial unit. This results hints at the possibility of model mispecification, and warrants a degree of caution in interpreting the model's outputs.

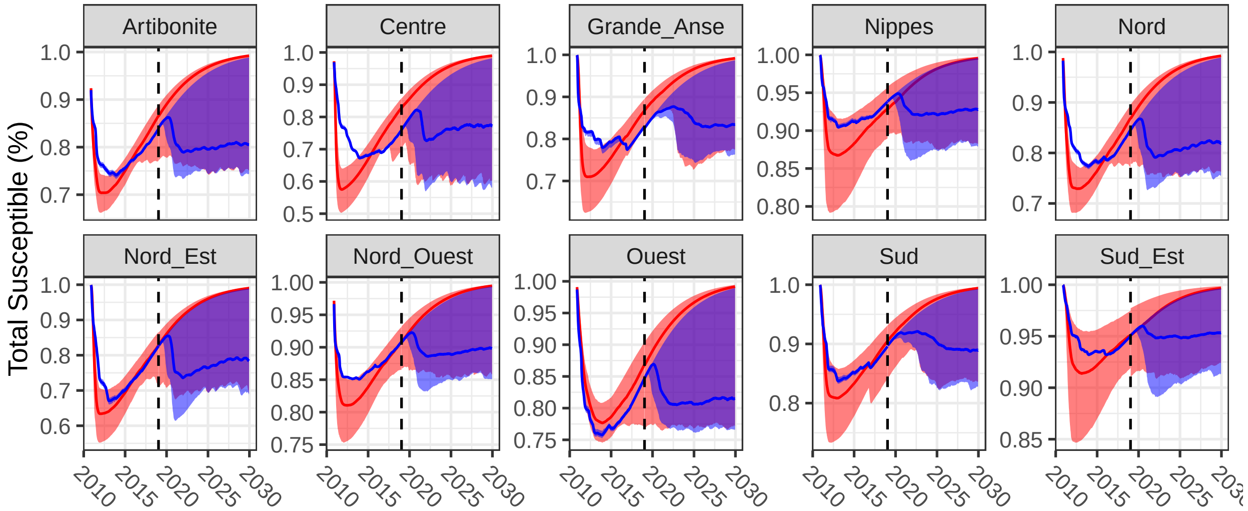

Fig S-5: Percentage of individuals that are in the susceptible compartment. Simulations from Model 3 under initial conditions are displayed in red; simulations from the filtering distribution of model are displayed in blue. The dashed line represents the end of the observed data.

References

[1] Lee EC, Chao DL, Lemaitre JC, Matrajt L, Pasetto D, Perez-Saez J, et al. Achieving Coordinated National Immunity and Cholera Elimination in Haiti Through Vaccination: A Modelling Study. The Lancet Global Health. 2020;8(8):e1081–e1089.

[2] Ionides EL, Ning N, Wheeler J. An Iterated Block Particle Filter for Inference on Coupled Dynamic Systems with Shared and Unit-Specific Parameters. Statistica Sinica, pre-published online. 2022;.

[3] Li J, Ionides EL, King AA, Pascual M, Ning N. Machine Learning for Mechanistic Models of Metapopulation Dynamics. arxiv:231106702. 2023;.

[4] Ferreira S. Cholera threatens Haiti after Hurricane Matthew. BMJ. 2016;355:i5516.

[5] Grad YH, Miller JC, Lipsitch M. Cholera Modeling: Challenges to Quantitative Analysis and Predicting the Impact of Interventions. Epidemiology. 2012;23(4):523.
