# Supplementary material for: Informing policy via dynamic models: Cholera in Haiti
Source: PLoS Comput Biol. 2024 Apr 29;20(4):e1012032. doi: 10.1371/journal.pcbi.1012032 (PMC11081515; doi:10.1371/journal.pcbi.1012032)
Supplement: S6 Text — A description of a empirical-Bayes approach that can be used to account for parameter uncertainty. (PDF) [file pcbi.1012032.s010.pdf]

Forecasting with parameter uncertainty

Let $f_{Y_{1:N}}(y_{1:N}|\theta)$ denote the pdf of the model under consideration, were θ is a parameter vector that indexes the model. Furthermore, denote the observed data as $y^*_{1:N}$. Because the uncertainty in just a single parameter can lead to drastically different forecasts [1], parameter uncertainty should be considered when obtaining model forecasts when the goal is to influencing policy. In a Bayesian modelling paradigm, the most natural way to account for parameter uncertainty in model forecasts is to suppose that θ comes from a distribution f_Θ, and then to obtain J forecasts from the model where each forecast is obtained using parameters drawn from the posterior distribution $\theta_{1:J} \mid Y_{1:N} = y^*_{1:N} \sim f_\Theta\big(\theta|Y_{1:N} = y^*_{1:N}\big)$.

When frequentist methods are used, however, there does not exist a posterior distribution from which one could sample. A common approach could be to obtain a weighted average of the simulations from various models [2], but this can be problematic when forecasts from each model are very different from each other [3]. A similar approach that has been taken [4] is to obtain model forecasts using multiple sets of parameter values and then sample from the resulting forecasts using weights proportional to the corresponding likelihoods of the parameter values. This approach could be considered as empirical Bayes, as it is equivalent to using a discrete uniform prior where the set of values in the prior distribution is determined by a stochastic routine applied to the observed data, as discussed below.

For each $k \in 1 : K$, let θ_k be a unique set of model parameters. Letting Θ denote a random vector of model parameters, we endow Θ with a discrete uniform distribution on the set $\{\theta_1, \theta_2, \dots, \theta_K\}$, such that $P\big(\Theta = \theta_k\big) = \frac{1}{K}$ for all values $k \in 1\!:\!K$. Using this as a prior distribution, the posterior distribution of $\Theta|Y_{1:N} = y^*_{1:N}$ can be expressed as: $P\big(\Theta = \theta_k|Y_{1:N} = y^*_{1:N}\big) = \frac{f_{Y_{1:N}}(y^*_{1:N}|\theta_k)}{\sum_{l=1}^K f_{Y_{1:N}}(y^*_{1:N}|\theta_l)}$. In a standard empirical Bayes analysis, the values $\theta_1, \dots, \theta_k$ of the prior distribution would be chosen using the observed data, resulting in a posterior distribution that weighs the prior parameter vectors proportional to their corresponding likelihoods. We choose θ_k to be the output of a stochastic routine applied to the observed data by setting θ_k to be the output of an iterated filtering algorithm. In practice, because the likelihood maximization routines of iterated filtering methods are stochastic, it is common to run the iterated filtering method multiple times (K) for each model in order to obtain a maximum likelihood estimate for model parameters. This results in a natural set of parameters near the MLE that could be used as the discrete prior distribution. Alternatively, the set $\{\theta_1, \theta_2, \dots, \theta_K\}$ could be determined by first obtaining marginal confidence intervals for each element of the parameter vector Θ, and then creating a hypercube using the combination of marginal confidence intervals. The set $\{\theta_1, \theta_2, \dots, \theta_K\}$ is then obtained by sampling uniformly K values from the resulting hypercube, as was done by [4].

References

[1] Saltelli A, Bammer G, Bruno I, Charters E, Di Fiore M, Didier E, et al. Five Ways to Ensure that Models Serve Society: a Manifesto. Nature. 2020;582:428–484.

[2] Hoeting JA, Madigan D, Raftery AE, Volinsky CT. Bayesian model averaging: a tutorial (with comments by M. Clyde, David Draper and E. I. George, and a rejoinder by the authors. Statistical Science. 1999;14(4):382 – 417. doi:10.1214/ss/1009212519.

[3] Grueber CE, Nakagawa S, Laws RJ, Jamieson IG. Multimodel inference in ecology and evolution: challenges and solutions. Journal of Evolutionary Biology. 2011;24(4):699–711. doi:https://doi.org/10.1111/j.1420-9101.2010.02210.x.

[4] King AA, Domenech de Cellès M, Magpantay FM, Rohani P. Avoidable Errors in the Modelling of Outbreaks of Emerging Pathogens, with Special Reference to Ebola. Proceedings of the Royal Society B: Biological Sciences. 2015;282(1806):20150347.
