# Supplementary material for: Informing policy via dynamic models: Cholera in Haiti
Source: PLoS Comput Biol. 2024 Apr 29;20(4):e1012032. doi: 10.1371/journal.pcbi.1012032 (PMC11081515; doi:10.1371/journal.pcbi.1012032)
Supplement: S7 Text — Tables and figures describing 95% confidence intervals for estimated parameters. (PDF) [file pcbi.1012032.s011.pdf]

Confidence Intervals for Model Parameters

In this section we provide confidence intervals for all model parameters, excluding those that take unique values for each spatial unit. For each model and parameter, we use principles of profile likelihood to obtain confidence intervals [1]. Due to the non-linear and stochastic nature of Models 1 and 3, exact evaluations of the profile log-likelihood are difficult to obtain. Instead, the log-likelihood at each point of the profile is estimated using via Monte-Carlo based particle filter methods. We therefore obtain confidence intervals for the parameters of Model 1 and Model 3 using the Monte Carlo adjust profile (MCAP) algorithm [2].

Profile confidence intervals for nonlinear POMP models are require a large number of computations. In the Model 1 and Model 3 subsections, we mention the total computational expense of each profile log-likelihood evaluation. Each subsection also provide figures that show the curvature of the profile log-likelihood near the MLE (Figures S-1–S-3). In these figures, the parameter values are shown on the transformed scale in which the profile was calculated.

S1 Model 1 parameters

Parameter estimates for Model 1, along with the MCAP confidence intervals for the estimate, are given in Table S-1. Figure S-1 displays the Monte Carlo evaluations of the profile likelihood values, obtained using a particle filter. The total computational burden of this profile likelihood search was 3631 hours, which was computed in parallel using 9675 separate jobs via the `batchtools` R package [3].

Table S-1: Model 1 parameter estimates and their corresponding confidence intervals, obtained via the MCAP algorithm.

**Mechanism**	**Parameter**	**MLE**	**95% Confidence Interval**
Seasonality	ζ	-0.036	$(-0.070, -0.008)$
Seasonality	β_1	1.417	$(1.277, 1.811)$
Seasonality	β_2	1.169	$(0.937, 1.445)$
Seasonality	β_3	1.136	$(0.990, 1.630)$
Seasonality	β_4	1.140	$(0.922, 1.389)$
Seasonality	β_5	1.401	$(1.261, 1.687)$
Seasonality	β_6	0.988	$(0.699, 1.132)$
Observation Variance	ψ : Epi	279.147	$(177.226, 990.191)$
Observation Variance	ψ : End	78.326	$(57.171, 204.654)$
Reporting Rate	ρ	0.679	$(0.315, 0.761)$
Mixing Exponent	ν	0.978	$(0.938, 0.999)$
Process noise ($\text{wk}^{1/2}$)	σ_{proc} : Epi	0.092	$(0.085, 0.113)$
Process noise ($\text{wk}^{1/2}$)	σ_{proc} : End	0.118	$(0.092, 0.179)$
Initial Values	$I_0(0)$	7298	$(2572, 1.4415 \times 10^4)$
Initial Values	$E_0(0)$	350	$(1, 1.3671 \times 10^4)$

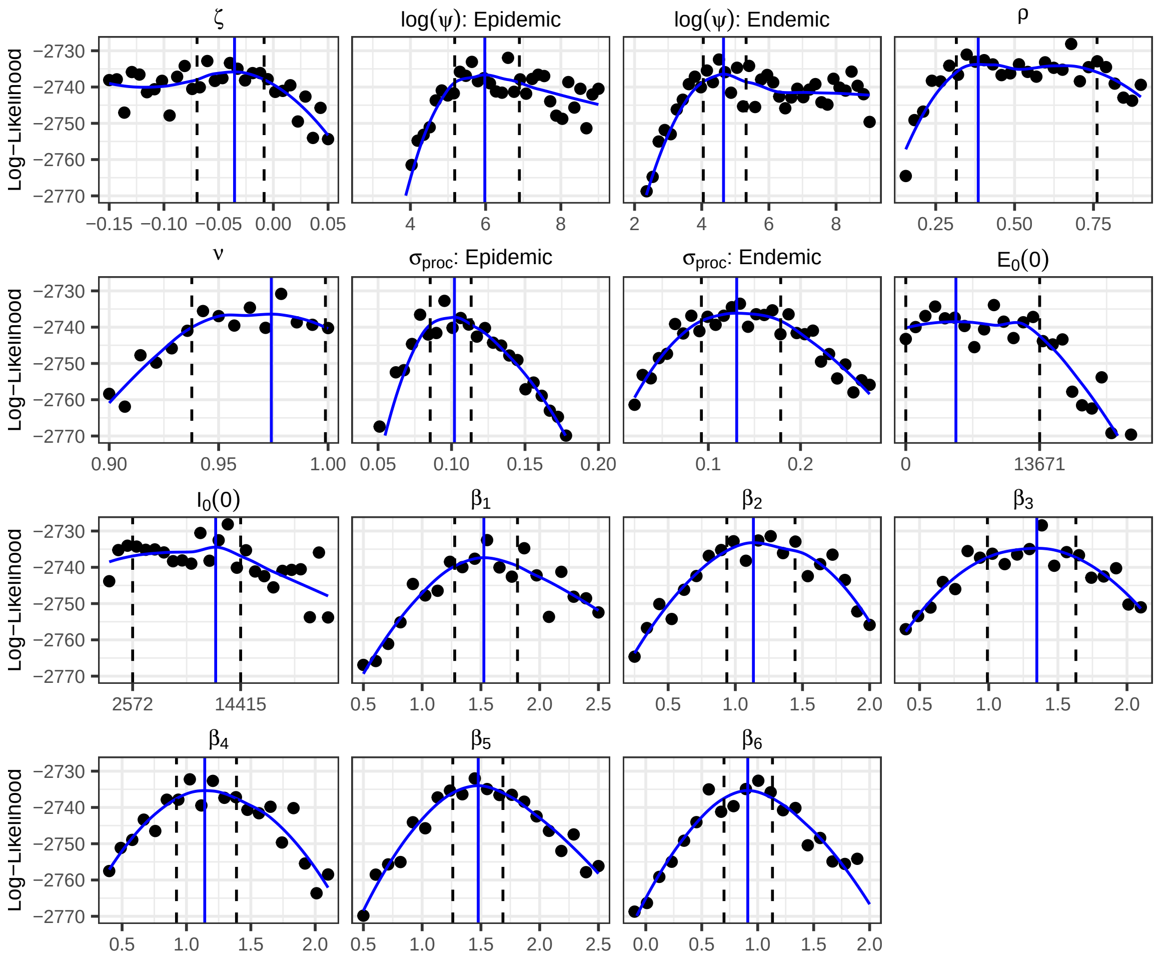

Fig S-1: MCAP confidence intervals for Model 1 parameters. The vertical blue line indicates the smoothed MLE.

S2 Model 2 parameters

Parameter estimates for Model 2, along with the profile likelihood confidence intervals for each estimate, are given in Table S-2. Figure S-2 displays the profile log-likelihood curve near the MLE. In Table S-2, the confidence interval for μ_{RS}^{-1}, the duration of natural immunity due to cholera infection, is arbitrarily large (going to infinity). This is possible because the parameter that was estimated was μ_{RS}, and the true MLE for this parameter is zero (see Figure S-2). This suggests that the fitted model favors a regime where reinfection events are not possible. Similarly, the MLE for the parameter β, which controls the amount of cholera transmission from human to human, is zero. Because Model 2 fails to describe the incidence data as well as a simple statistical benchmark, we must be careful to not interpret these results as evidence that reinfections and human-to-human infection events do not occur. Instead, we may consider this as additional evidence of model mispecification.

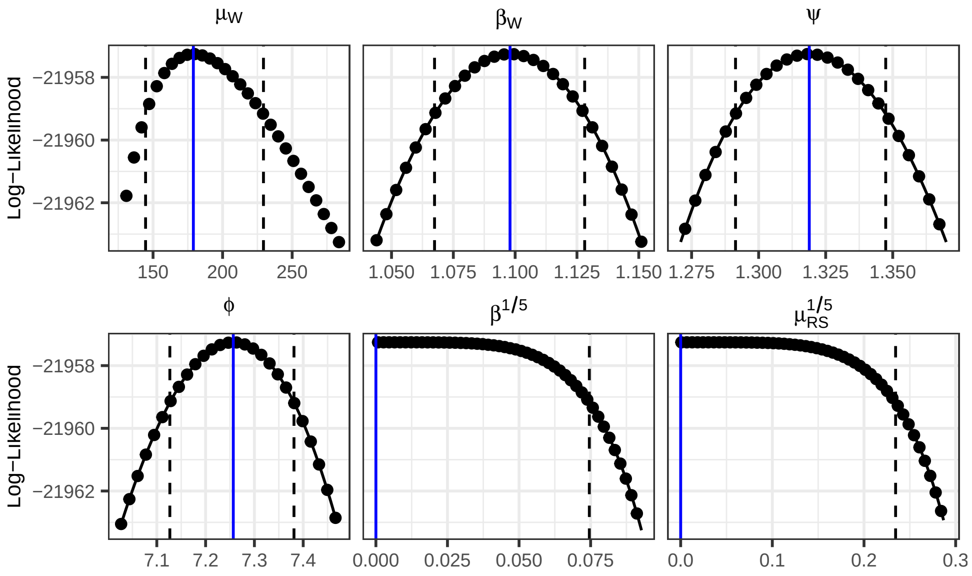

Fig S-2: MCAP confidence intervals for Model 2 parameters. The vertical blue line indicates the MLE.

Table S-2: Model 2 parameter estimates and their corresponding confidence intervals, obtained via profile likelihood.

**Mechanism**	**Parameter**	**MLE**	**95% Confidence Interval**
Human to water shedding (wk^{-1})	μ_W	179.2	$(144.6, 229.4)$
Water to Human Infection (yr^{-1})	β_W	1.098	$(1.067, 1.128)$
Observation Variance	ψ	1.319	$(1.291, 1.347)$
Seasonality	ϕ	0.974	$(7.127, 7.381)$
Human to Human Infection (yr^{-1})	β	$5.97 \times 10^{-15*}$	$[0, 2.3 \times 10^{-6})$
Immunity (yr)	μ_{RS}^{-1}	$1.4 \times 10^{11*}$	$(1410, \text{inf})$

*As evident in Figure S-2, the true MLE for these parameters is 0 and ∞, respectively; this value could not be obtained numerically due to the parameter transformation applied to the parameter for the model fitting processes.

S3 Model 3 parameters

Parameter estimates for Model 3, along with the MCAP confidence intervals for the estimate, are given in Table S-3. Figure S-3 displays the Monte Carlo evaluations of the profile likelihood values, obtained using a particle filter. The total computational burden of this profile likelihood search was 28938 hours, which was computed in parallel using 7568 separate jobs via the `batchtools` R package [3].

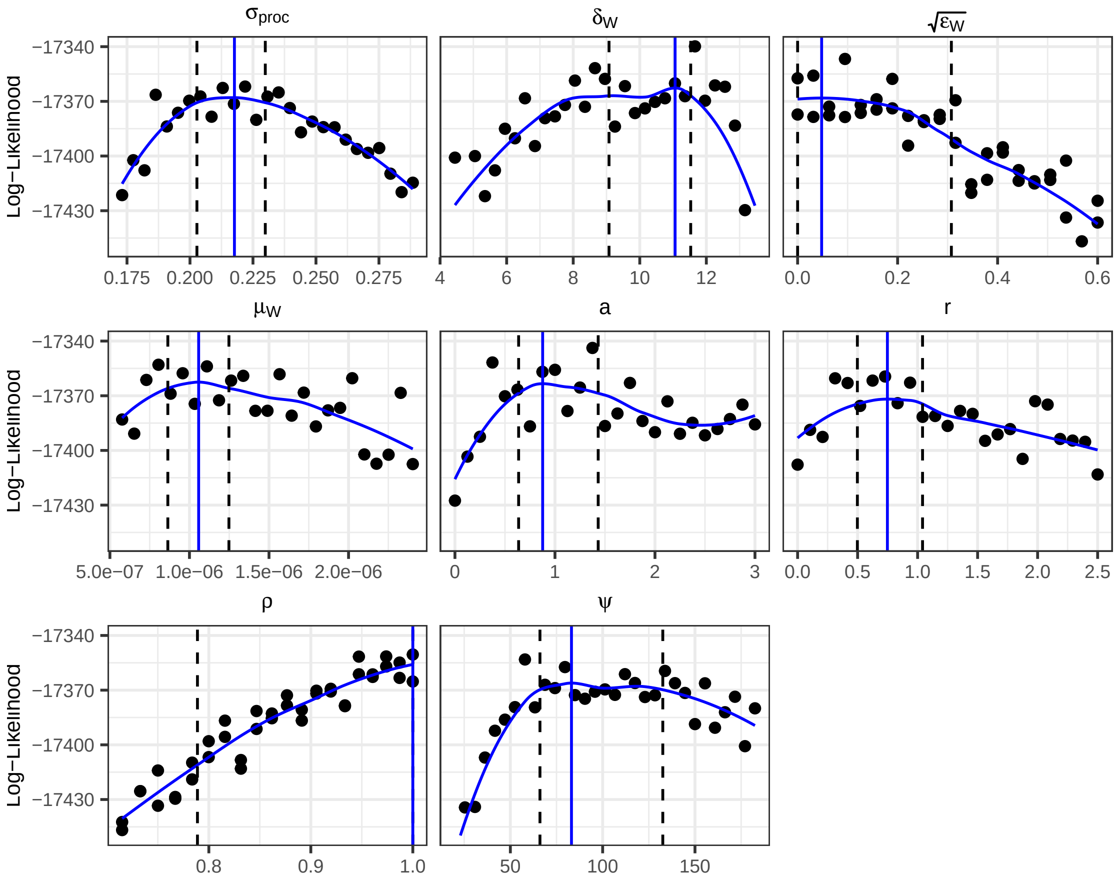

Fig S-3: MCAP confidence intervals for Model 3 parameters. The vertical blue line indicates the smoothed MLE.

Table S-3: Model 3 parameter estimates and their corresponding confidence intervals, obtained via the MCAP algorithm.

**Mechanism**	**Parameter**	**MLE**	**95% Confidence Interval**
Process Noise ($\text{wk}^{1/2}$)	σ_{proc}	0.218	$(0.203, 0.230)$
Water Survival (wk)	δ_W^{-1}	0.108	$(0.087, 0.110)$
Human to Water Shedding $\frac{\text{km}^2}{\text{wk}}$	μ_W	9.77×10^{-7}	$(8.64 \times 10^{-7}, 1.25 \times 10^{-6})$
Asymptomatic Shedding	ϵ_W	0.008	$(0.0, 0.095)$
Seasonality	a	1.000	$(0.637, 1.432)$
Seasonality	r	0.780	$(0.498, 1.041)$
Reporting Rate	ρ	0.983	$(0.789, 1.000)$
Observation Variance	ψ	88.578	$(66.034, 132.563)$

References

[1] Pawitan Y. In all likelihood: statistical modelling and inference using likelihood. Oxford University Press; 2001.

[2] Ionides EL, Breto C, Park J, Smith RA, King AA. Monte Carlo Profile Confidence Intervals for Dynamic Systems. Journal of the Royal Society Interface. 2017;14:1–10.

[3] Lang M, Bischl B, Surmann D. batchtools: Tools for R to work on batch systems. The Journal of Open Source Software. 2017;(10).
