# Supplementary material for: Informing policy via dynamic models: Cholera in Haiti
Source: PLoS Comput Biol. 2024 Apr 29;20(4):e1012032. doi: 10.1371/journal.pcbi.1012032 (PMC11081515; doi:10.1371/journal.pcbi.1012032)
Supplement: Attachment — Submitted filename: response.pdf [file pcbi.1012032.s013.pdf]

March 4, 2024

Re: PCOMPBIOL-D-23-01609. "Informing policy via dynamic models: Cholera in Haiti"

Dear PLOS Computational Biology editorial board,

Thank you for arranging the review of our manuscript, and for the invitation to submit a revision. We are grateful for the thoughtful comments from the referees. Our revised manuscript has various edits for clarity, following the referee suggestions, as well as some small corrections in the text.

One of the reviewers recommended the inclusion of confidence intervals for model parameters, which was not originally included because the paper that we re-analyze did not include them. However, we agree that the inclusion of confidence intervals is beneficial for readers, so we have done so in the revised manuscript. The additional computational effort required to obtain confidence intervals resulted in slightly improved parameter estimates, though qualitative conclusions drawn from the fitted models remain unchanged.

The other main change in the revised manuscript is edits to the introduction and discussion, as recommended by both reviewers. These changes help make the goals of the mansucript clear, and connect the specific case study that was conducted to these goals. This resulted in improvements to the article without modifying the overall content of the original submission.

Below are point-by-point responses to the referee reports. The reviewer comments are shown in green, italic type. Our responses are in black, and material copied verbatim from the revision is in blue.

Sincerely,

Jesse Wheeler
PhD Student, Statistics Department
University of Michigan
jeswheel@umich.edu

Referee 1

The authors' manuscript aims to explain how public health decisions can best be informed by combining an understanding of the underlying pathogen transmission dynamics with epidemiological data. In particular, the authors make a number of recommendations of best practices that modellers can adopt when using mechanistic models to make statistical inferences from time series data. To make their recommendations more concrete, they perform a re-analysis of cholera incidence data from Haiti spanning the years 2014-2019. These data were previously analysed in a modelling study performed by a separate team of scientists, published in Lee et al. (2020), see Ref. 1. Using refinements of three models from Lee et al., the authors demonstrate a number of ways in which the analysis could have been improved. Furthermore, the authors present differences between the findings of their reanalysis and the original analysis of Lee et al., for instance in the predicted probability of eliminating cholera from Haiti (see Figure 8).

My most substantial concern is with the framing of the paper, and whether that might mislead potential readers. To me, the paper serves more of an educational purpose, presenting ideas on how to best use mathematical models to gain insight from time series data. While the case study into cholera in Haiti is methodologically sound, it does not seem to be the main focus of the manuscript. To such an extent that, in the discussion, the only comment to on the Haiti analysis is that the authors "used the same data and models, and even much of the same code, as Lee et al. [1], and yet ended up with drastically different conclusions."

This major concern can be resolved in two ways, both of which seem acceptable to me, either a) reframe the paper as a more general series of recommendations for how to better perform statistical inference on time series data using mechanistic models (including a longer introduction and discussion of existing methodological deficiencies) or b) perform a more in-depth analysis and commentary on cholera in Haiti (some of which has already been done but is buried in supplementary information files that are only briefly mentioned in the main text, e.g. S5).

Thanks for the feedback on the framing of our contribution. The original goal of our manuscript was to combine the two foci identified by the referee: we use an in-depth case study to motivate general procedures for fitting mathematical models to time series data, while explaining how these procedures help to address limitations of previous approaches. The case study was intended both to help motivate the advice and to make sure that the advice is pertinent and practical on at least one suite of models. Since our approach is predicated on the possibility of carrying out some nontrivial computational procedures, we are led to address computational issues in parallel with scientific and statistical issues.

The referee would prefer us to emphasize one of these foci, and this feedback has helped us see how readers might find themselves struggling to orient themselves while reading the manuscript. We agree that the "educational" purpose of the paper is primary to the specific conclusions about the Haiti cholera epidemic. However, the validity of our reanalysis of the Haiti data and models provides critical support to our methodological points. For the revision, we have edited the abstract and introduction to make explicit how the case study fits into the overall goals of the paper. In the discussion, we return from the concrete consideration of the case study, placing it back in the

more general setting of inferring complex dynamics from time series data. In the revision, we have added more signposts to help the reader see how the points that arise in the discussion generalize the corresponding points arising in the case study.

> *I found the methodology used to be well-suited to addressing the aims of the paper.*
>
> *Overall, I found the paper interesting and informative, but a little unfocused. I expect with some heavy rewriting in places (especially the introduction and discussion), the paper will be acceptable for publication in PLoS Computational Biology. I do not anticipate the authors having to perform additional computational analysis.*

Thank you for this feedback. Our primary changes in the revised manuscript include edits to the abstract, author summary, and introduction, with the goal of making the focus of the paper more clear. We have also done a nearly complete rewritting of the discussion section, improving the connection between the our overal goals with the cholera case study. Specifically, though the content and arguments made in the discussion remain the same, we point out ways that our case study demonstrates the usefulness of our general suggestions for model based inference of dynamic systems.

> ***Abstract***
> *The authors state they "develop data analysis strategies leading to improved statistical fit." It's not clear to me exactly what novel strategies have been developed, it seems the authors exclusively use pre-existing techniques from the literature. This is fine, if the focus of the paper is either a) pedagogical or b) on understanding a specific disease system (cholera in Haiti). If any methods are novel, this should be more clearly stated.*

Fitting models 1 and 2 does not require any methodological innovation. Model 3 has a stochastic spatiotemporal partially-observed structure that leads to methodological challenges. [1] made some approximations to avoid carrying out inference for the full coupled system. We show some consequences of those approximations, taking advantage of a newly developed method which has not yet been applied in the context of scientific investigations. So, although the method is not entirely new, its applicability to this task is novel. Additional commentary to this point has been added in the abstract, introduction, and discussion sections.

We have further modified the statement in the abstract that led to this confusion. Rather than stating that we "develop data analysis strategies leading to improved statistical fit", we now say:

> *We assess current methodological approaches to these issues via a case study of the 2010-2019 cholera epidemic in Haiti. We consider three dynamic models developed by expert teams to advise on vaccination policies. We evaluate previous methods used for fitting these models, and we demonstrate modified data analysis strategies leading to improved statistical fit. Specifically, we present approaches for diagnosing model misspecification and the consequent development of improved models. Additionally, we demonstrate the utility of recent advances in likelihood maximization for high-dimensional nonlinear dynamic models, enabling likelihood-based inference for spatiotemporal incidence data using this class of models.*

Introduction

1. To make clear that the issues being addressed by this paper go beyond one modelling study of Haiti, the authors should provide examples and references to support the (accurate in my opinion) statement about, "common modeling decisions that may not provide an adequate statistical explanation of the data".

That statement arose in the author summary, where references are not expected. However, we appreciate that the point should be expanded on, and we have done so in the revision. Specifically, we cite papers from the literature review we performed on the use of dynamic models for analyzing the Haiti cholera oubreak. Our literature review was previously mentioned, but citations from the review were not made. Furthermore, we modified the quoted statement in the author summary to be the following:

In this article, we conduct a re-analysis of the 2010-2019 cholera outbreak in Haiti. We use this case study to investigate current procedures for fitting mechanistic models to time series data, while identifying limitations of these methodologies and proposing remedies.

Among the most common mistakes in mechanistic modeling, in our opinion, is not critically evaluating the statistical fit of the model. We originally tried to make this point by having an entire section dedicated to model diagnostics, though we admit that the connection between the statement in the author summary and a section later in the article was not clear. We have addressed this by adding sign-posts to references and suggestions back to this statement, and other similar statements in the abtract / introduction. Our modified discussion also includes detailed examples of principles that were very useful in our case study but are not yet universal practice.

2. Given the focus of the paper on how to better perform inference, explicitly commenting on what are the "existing guidelines for creating models to inform policy [4, 5]". It would then also be helpful in the discussion to comment on how the recommendations of this study extend these pre-existing guidelines.

We have added a paragraph in the introduction discussing how recommendations we make in this article fit in the broader category of papers that make recommendations on fitting mechanistic models to dynamic systems. This includes discussing these two papers that we originally cited in more detail, as well as other articles on similar topics:

Numerous guidelines have been proposed for using mechanistic models to inform policy, reviewed in [2]. Behrend et al. [2] identify the importance of stakeholder engagement, transparency, reproducibility, uncertainty communication, and testable model outcomes. These and related principles are echoed by other influential articles [3, 4]. Additional literature emphasizes model calibration and evaluation techniques [5–7]. These guidelines often lack implementation specifics. As an example, [1] largely adhere to the principles of [2]—though assessing the extent of stake-holder engagement is challenging—yet their projections are inconsistent with actual cholera incidence data from 2019 to 2022, demonstrating the limitations of current standards. We provide methodology for rigorous statistical calibration and evaluation of dynamic models (as advocated by [8]), thereby expanding on the prevailing guidance. We specifically emphasize principles that prove essential in our case study. Complementary methodological suggestions arising from a spatio-temporal analysis of COVID-19 are detailed in [9].

We have also added some material in the discussion that connects our results to the stated goals

of the paper in the abstract and introduction.

Methods

3. Fig 2. Model parameters. I think this should be "Table 1"?

While Fig 2 really is a table, here we are following PLOS Computational Biology Submission guidelines (`https://journals.plos.org/ploscompbiol/s/tables`):

> "If the table has a very complex structure or contains graphics, the safest solution is to make it into a figure. Export the table as a TIFF, and cite and re-label it as a figure."

The Table in this case is necessarily complex, as we are trying to put all comparable model parameters from three seperate models into a single table. If the journal requests that we make this into a table rather than a figure we are happy to comply.

4. "and $z \in 1 : Z$ describes hypothetical vaccination programs" It would help to give an example of the different vaccine programs here. E.g. does this mean the number of doses the individual has received, or differences in the vaccine administered?

It could be either of the above, the main point being that the subscript seperates individuals into compartments that have distinct dynamics. We clarified this by adding the following sentences:

> *Here, $z = 0$ corresponds to unvaccinated individuals, and $z \in 1 : Z$ describes hypothetical vaccination programs. Each program z indexes differences in both the number of doses administered (one versus two doses per individual) and the round of vaccine administration, seperating individuals into compartments with distinct dynamics based on vaccination status.*

In our analysis we only consider vaccination dynamics based on the scenarios outlined in the Forecasts section. For Model 1, the assumption made by Lee et al. [1], and reused in our analysis, is that the added dynamics of the vaccination compartments is an increased probability that an infection is asymptomatic, as described in an unmodified paragraph that follows the quoted text.

5. Eq 6 and line 1: I think the notation for the asymptomatic fraction might be clearer and more consistent with the other models if you used $f_z(t)$ instead of $\vartheta_z(t)$. In fact, this is the notation used in the table in Figure 2.

Thank you for this suggestion, we have made this change.

6. Line 198: Reference for where value of v_{rate} comes from.

This parameter comes from Lee et al. [1], and the only details that they provide is a subscript comment in one of their tables (S13) in their supplement: "v_{rate} was chosen to mimic the distribution of infections in Haiti". In the same table, however, they do not list v_{rate} as a parameter that was "fit", making it unclear where the value comes from. Presumably, it was chosen somewhat subjectively based on their understanding of cholera dynamics in Haiti, because in order to obtain a fit for their model they had to have fixed the value at something without actually estimating it, and perhaps only considered a very small set of possible values. Notably, this parameter can mathematically be estimated like all other parameters in the model, but their specific implementation of Model 2 does not permit its estimation. A full discussion on parameters that are partially estimated via subjective measures is outside the scope of our article. To clarify where this fixed value comes from the revised manuscript reads:

$v_{\text{rate}} = 10^{-12}$ km^{2}yr^{-1} was fixed at the value used in [1].

7. Usually, vaccine efficacy = 1 implies the vaccine completely blocks transmission and 0 implies no effect. In model 2 the definition of vaccine efficacy (lines 200-205 and Eq 14) implies the opposite. I suggest re-parameterising the model, or renaming the variable. For what its worth, models 1 and 3 seem to follow the usual convention.

This change has been made.

8. I found the commentary on comparing fitted mechanistic models to a (statistical) benchmark model to be informative and helpful. I was wondering if the authors had any comments on whether (or not) to directly compare the AIC values of the different fitted models directly. Am I right in thinking this should be possible for models 2 and 3 given (I think) they are fitted to the same data? Model 1 is fitted to a different data set, which I understand makes such a comparison impossible.

It is correct that models fit to the same datasets can be directly compared using the AIC values. Models 2 and 3 are not fit to the same datasets, however, due to the way Model 3 is initialized. Details on how they are initialized are provided in the section with the model descriptions, as well as supplement S5.

We added the following sentence to clarify this point:

Models that are fit to the same datasets can be directly compared using AIC values, making it a useful tool to compare to benchmark models. Though Models 2 and 3 are both fit to department level incidence reports, their AIC values are not directly comparable due to the way Model 3 initializes latent states (S2 Text).

9. Lines 308-309: Isn't this a log-linear trend in the transmission rate?

Yes, that is correct. This has been fixed in the revision. In other instances where the linear trend was previously mentioned, we simply refer to it now as a trend in transmision rate, except where the additional detail of the trend being log-linear is needed for clarity.

10. While the data support a linear trend at the 95% confidence level, it's worth commenting on the magnitude of the trend, e.g. the total reduction in $\beta(t)$ over the period of study.

Thank you for this suggestion, a sentence on the overall effect of the parameter estimate has been included in the revised manuscript:

The maximum likelihood estimate for ζ corresponds to a 7.3% reduction to the transmission rate over the course of the outbreak, with a 95% confidence interval of $(1.8\%, 17.9\%)$ for the overall reduction in transmission.

11. I might have misunderstood it slightly, but I don't entirely follow the reasoning of lines 340-346. It seems that two different claims are being made: A) the model can't identify which mechanism underlies a trend in $\beta(t)$ and B) the model can't definitively state there is a trend in $\beta(t)$ compared with some other time-varying parameter (e.g. reporting probability). For model 1, I agree with the authors that claims A and B are valid. What I take issue with is the final statement, which seems to be stronger and apply to both claims and mechanistic models more generally: "we argue that a decreasing transmission rate is a plausible way to explain this, but the incidence data themselves do not provide enough information to pin down the mechanism." I agree that the "incidence data themselves" (which I take to imply "without additional covariates data") make claim A valid regardless of the model. However, I don't think claim B holds regardless of the model. By changing model 1 to include time variation in the reporting rate I don't see a priori *why the "the incidence data themselves" might not provide enough information to distinguish between time variation in transmission compared with time variation in reporting without the need for additional data. The deficiency is in model 1 rather than the information content of the data. I suggest the authors rework the paragraph to make the reasoning clearer.*

Our views align with the referee's comments, and we have rewritten the article to make this clearer. The revised paragraphs with this statement are now:

If a mechanistic model including a feature (such as a representation of a mechanism, or the inclusion of a covariate) fits better than mechanistic models without that feature, and also has competitive fit compared to associative benchmarks, this may be taken as evidence supporting the scientific relevance of the feature. As for any analysis of observational data, we must be alert to the possibility of confounding. For a covariate, this shows up in a similar way to regression analysis: the covariate under investigation could be a proxy for some other unmodeled phenomenon or unmeasured covariate.

The statistical evidence of a trend in transmission rate in this model could be explained by any trending variable (such as hygiene improvements, or changes in population behavior), resulting in confounding from collinear covariates. Alternatively, it is possible that the negative trend observed in the incidence data could be attributed to a decreasing reporting rate rather than decreasing transmission rate. This could be formally tested by comparing models with either trend specification. We did not do this because evidence suggests that reporting rate was maintained or increased (Figure 1 of [10]). We instead argue that a decreasing transmission rate is a plausible way to explain the decrease in cases over time, as there is alternative evidence that supports this model [10–12]. It is not practical to test all remotely plausible model variations, yet a strongly supported conclusion should avoid ruling out untested hypotheses. The robust statistical conclusion for our analysis is that a model which allows for change fits better than one which does not, and a trend in transmission is a plausible way to do this.

12. Lines 553-556: I push back against this point. The models assume an exponential distribution for vaccine-derived immunity. Assuming a mean duration of 10 years, the proportion of individuals who remain immune 9 years after vaccination is . Even for much larger durations of immunity a non-negligible fraction of the population will lose immunity after 9 years, e.g. $e^{-9/10} \approx 0.41$*. I therefore don't expect values for the duration of immunity around 10 years to "effectively result in the same model dynamics".*

The qualitative comparison we had in mind was the regime with short immunity where many people become infected multiple times during the ten year epidemic, allowing multiple epidemic peaks due to susceptible replenishment. We have reworded the sentence to avoid making too strong a claim about 10yr immunity being a precise point where the dynamics transition to a regime where reinfection events become negligible. The revised paragraph is provided below.

> *Our case study provides an example of this in the parameter estimate for the duration of natural immunity due to cholera infection, μ_{RS}^{-1}. Under the framework of Model 2, the best estimate for this parameter is 1.4×10^{11} yr, suggesting that individuals have permanent immunity to cholera once infected. Rather than interpreting this as scientific evidence that individuals have permanent immunity from cholera, this result suggests that Model 2 favors a regime where reinfection events are a negligible part of the dynamics. The depletion of susceptible individuals may be attributed to confounding mechanisms—such as localized vaccination programs and non-pharmaceutical interventions that reduce cholera transmission [10, 13]—that were not accounted for in the model. Perhaps the best interpretation of the estimated parameter, then, is that under the modeling framework that was used, the model most adequately describes the observed data by having a steady decrease in the number of susceptible individuals. The weak statistical fit of Model 2 compared to a log-linear benchmark (see Table 2) cautions us against drawing quantitative conclusions from this model. A model that has a poor statistical fit may nevertheless provide a useful conceptual framework for thinking about the system under investigation. However, a claim that the model has been validated against data should be reserved for situations where the model provides a statistical fit that is competitive against alternative explanations.*

Discussion

13. In contrast with the introduction, which almost exclusively focuses on cholera in Haiti, the discussion only mentions it once, briefly: "We used the same data and models, and even much of the same code, as Lee et al. [1], and yet ended up with drastically different conclusions." It would help the reader to have those discrepancies summarised. It would be helpful to summarise the recommendations the authors make attempting to fit mechanistic models to time series data (even as a list). Along similar lines, it would be helpful to present a more concise summary of what the "more accurate policy evaluations" found by using the approaches outlined in this study.

The revised version of the paper contains significant edits to the introduction and discussion sections that we feel address this concern. In the discussion, the revised mansucript specifically mentions how the recalibrated models suggest a moderately high probability of cholera elimination from 2019-2022, which is consistent with what actually occured. We also discuss scientific insights that can made with the recalbrated models, such as the effect of Hurricane Matthew on cholera incidence, the importance of rainfall as a driver of cholera infection in Haiti, and the presence of overdispersion in the cholera dynamics.

Typos

Eq. 1: Is there a missing star on $y_{1:N}$ on the right-hand-side?

Yes, there should be an asterisk. This has been fixed.

Line 146: delete "the" before describing

Fixed.

Referee 2

This well-written article is a strong piece of work that will be useful to the readers of this journal and, in particular, to researchers who perform statistical inference on compartmental models of infectious diseases. Furthermore, this paper is enhanced by its transparency and reproducibility, as the provided code is well-documented and organised in an R package. Additionally, the supplementary information is a valuable resource for researchers in this field.

In a nutshell, the authors argue that existing criteria to evaluate the validity of a disease model are insufficient. Therefore, they propose more stringent standards for evaluating models' ability to fit the available data in order to obtain more reliable forecasts. The authors use a Cholera case study to outline their suggestions. To highlight, a key contribution from this work is the recommendation of employing inductive (associative) models as a goodness-of-fit benchmark, as evidenced by this sentence: "It should be universal practice to present measures of goodness of fit for published models, and mechanistic models should be compared against benchmarks". Undoubtedly, this approach provides an objective measure to judge the ability of mechanistic models to fit the data. In the following sections, I express my opinion on how this paper may be improved.

Major Concerns

1. Literature

While the arguments provided throughout the article are well-articulated, there needs to be more supporting literature at the beginning of major sections. For instance, I don't need to be convinced that the structure of a model should be based on a realistic theory about the observed phenomenon; namely, models should be a white box. However, not everyone is on board with this premise, and supporting literature that argues in favour of this approach should be mentioned. In short, more citations should be added at the beginning of each major section.

The referee's suggestions have led to the addition of various references in the revision, which now includes over 100 citations. For the particular issue of black box versus white box models, we have included the following paragraph:

> *Mechanistic models representing biological phenomena are valuable for epidemiology and consequently for public health policy [14, 15]. More broadly, they have useful roles throughout biology, especially when combined with statistical methods that properly account for stochasticity and nonlinearity [16]. In some situations, modern machine learning methods can outperform mechanistic models on epidemiological forecasting tasks [17, 18]. The predictive skill of non-mechanistic models can reveal limitations in mechanistic models, but cannot readily replace the scientific understanding obtained by describing the biological dynamics of the system in a mathematical model [18, 19].*

This addition is intended to compliment the discussion about mechanistic (white-box) vs associative (black-box) models that was part of the "Materials and methods" section.

2. Limitations

In various passages of this paper, it is hinted that modellers should refrain from deterministic models and instead opt for more realistic stochastic representations. While the critique of ODE models is valid, the shift to stochastic structures is not a free choice. For example, introducing extra-demographic variability adds one additional parameter (infinitesimal variance). In ODE models, one extra parameter can lead to unidentifiability, and there's no apparent reason why this would differ in a stochastic version. Moreover, transitioning to stochastic models involves abandoning well-established MCMC algorithms in favour of methods still in development. Therefore, modellers should not assume that more realistic models with additional parameters are necessarily better without proper caution. In my experience, diagnosing unidentifiability is easier in ODE models than in POMP structures. Hence, there is a trade-off between benefits and costs.

The referee raises several points in this paragraph, and we consider them one at a time.

Moving from an ODE model to a stochastic model may not involve any additional parameters, if only demographic stochasticity is included. We agree, however, that adding to model complexity has costs, including loss of identifiability. In our revised manuscript, we address identifiability via profile likelihood methods (S7 Text).

In our study, we find that the deterministic model (model 2) has poor statistical fit (Table 2). A poor fit can lead to a risk of identifying spurious scientific phenomena: additional deterministic complexity added to the model may help to fit the model to unmodeled stochastic phenomena present in the data, and may therefore spuriously be assessed as a highly statistically significant model improvement. We see some evidence of this in our analysis. Aside from not being stochastic, the mechanistic structure of Model 2 is more complex than the other two models considered in our study. Model 2 also demonstrates the most clear cases of spurious scientific findings, as some estimated parameters favor regions of the parameter space that we would not expect scientifically—such as permanent immunity from infection once recovered from an infection (μ_{RS}^{-1}), and zero human-to-human transmission (β). This may partially explain the choice of Lee et al [1] to consider a scientifically unjustifiable jump in latent state values from their epidemic to endemic periods: without this additional flexibility, Model 2 cannot adequately describe cholera dynamics in Haiti while keeping parameter values in scientifically plausible ranges. From our experience, this type of a result is quite common: for a deterministic model to adequately account for unmodeled features of a dynamic system, parameters that are estimated are often forced to unexpected regions of the parameter space.

Adding stochasticity to the model helps address this issue by capturing unmodeled features in the inherent stochasticity of the model rather than in estimated parameters. Stochasticity in the model alone may not fully solve this problem as evidence suggests that the failure to account for extra-demographic variablility may lead to biases in parameter estimates [20]. Our revision includes the addition of profile likelihood for model parameters, which also provide strong evidence for the inclusion of extra-demographic stochasticity in Models 1 and 3 (see supplement material section S8). Generally, it is advisable to consider the possibility of over-dispersed variation on the transmission rate [20, 21] leading to one extra parameter compared to a deterministic model. In the context of cholera dynamics, [22] found a substantial improvement in model fit by including stochasticity, involving one over-dispersion parameter. [23] found similar results, favoring a stochastic dynamic model with over-dispersion, when investigating transmission dynamics of a dif-

ferent enteric pathogen, rotavirus. In other contexts, [24] noted various scientific advantages for including stochasticity in the model; it is undoubtedly part of the biological dynamics, and its omission can lead to over-confident predictions. Our revised manuscript includes several references demonstrating the importance of modeling extra-demographic stochasticity, including our revised discussion:

> *Unmodeled features of a dynamic system can lead to spurious or misleading parameter estimates if the features substantially impact observed data. In deterministic models, features that cannot be explained by measurement error must be accounted for by the choice of parameters. For our case study, some of the parameter estimates for the deterministic Model 2 are implausible, such as the infinite immunity discussed above, and this may be explained by compensation for model misspecification. Incorporating demographic and environmental stochasticity into models can mitigate the impact of unmodeled features. Stochastic phenomena are not only arguably present in biological systems, but their inclusion in a model also allows observed data variations to be attributed to inherent uncertainty rather than to distorted parameter values. Models 1 and 3 suggest the presence of extra-demographic stochasticity [9, 20, 23], as evidenced by the confidence intervals for the corresponding parameter σ_{proc} (S7 Text).*

The decision on whether to choose Markov chain Monte Carlo (MCMC) methods is not fully aligned with the decision on whether to include stochasticity in the model. In our literature review, of the 12 previous Haiti cholera investigations including stochastic dynamic models [1,25–34], four of them carried out inference via MCMC [26, 29, 32, 33]. We agree with the referee that MCMC is more routine for determinstic models than for stochastic models, and advanced MCMC methods such as Hamiltonian Monte Carlo become more readily applicable. Sequential Monte Carlo (SMC) was used for only two of the investigations in our literature review [1, 34]. In principle, MCMC methods can be applied for inference in over-dispersed stochastic dynamic models via so-called particle MCMC (PMCMC) [35]. In practice, these models have been fitted by maximum likelihood, some examples including [22, 23, 36–39].

Despite their inability to describe incidence data, deterministic models can do a good job at detecting overal trends, like the gradual decline of cholera cases in Haiti. This, coupled with the ease in fitting ODEs to data, makes ODEs a useful tool to gain preliminary insight on a dynamic system. They are also a useful tool to understand a stochastic model [40]. Because of their usefulness, we do not go as far as to suggest that deterministic models should not be used. Our revised discussion includes a comment that demonstrates this belief:

> *Despite their limitations, deterministic models can offer valuable insights into dynamic systems [16]. In [1], the forecasts from the deterministic Model 2 were qualitatively more consistent with the observed disappearance of cholera than the stochastic models.*

In summary, maximum likelihood (ML) inference via SMC nowadays provides a fairly well estabilished alternative to Bayesian MCMC inference. ML appears to be methodologically more convenient for stochastic dynamic models with overdispersion. When analyzing long epidemiological datasets, this stochasticity appears to be important for statistical model fit. That alone is not enough to show that models with overdispersed stochastic dynamics are scientifically preferable. However, discovery of a model feature which generates a large improvement in statistcal fit from adding one more parameter is typically treated as scientific evidence favoring the inclusion of that

feature in a scientific model for the phenomenon.

> *Moreover, Monte Carlo methods, such as the Particle Filter and, by extension, Iterated Filtering, aim to approximate integrals (the posterior or filtering distributions). However, one cannot take for granted that these methods provide accurate descriptions of these targets without proper validation. In more 'traditional' MCMC methods, diagnostics like the potential scale reduction factor and effective sample size play a crucial role in the inference process. Unsatisfactory values of these diagnostics render inferences unreliable, often necessitating model reformulation. In contrast, in the literature on POMP models (including this paper), diagnostics are tangentially mentioned. Users (like me) sometimes face uncertainty about whether the lack of fit is due to model misspecification or problems with the Monte Carlo algorithm exploring the parameter space.*

We agree with the referee that diagnostics are an important aspect of careful data analysis. The most widely used diagnostic tools for particle filter and iterated particle filter methods probably match those provided by default in the `pomp` R package [41] when objects generated by `pfilter()` or `mif2()` are plotted. For filtering, one tracks the effective sample size, the conditional log likelihood for each observation, and the values of the filtered states. For iterated filtering, one checks the filtering diagnostics for the last iteration and also how the parameter estimates and the log-likelihood estimate evolve through iterations, typically superimposing the results of 10-100 independent searches.

The particle filter provides access to the likelihood of the data, and conditional likelihood of each individual data point. By contrast, obtaining reliable likelihood values from MCMC is not straightforward. Thus, particle filter methods facilitate the use of various likelihood-based methods for diagnostics and model selection. We demonstrate this in the supplement (S5 Text, S7 Text).

As a proper scoring rule [42], log-likelihood can be used as an effective way to evaluate filters as well as models. The particle filter is unbiased for the likelihood, and therefore negatively biased for the log-likelihood with bias approaching zero as the number of particles increases and the Monte Carlo variance decreases. If optimization searches from diverse starting points provide a consensus on the maximum log-likelihood, and the Monte Carlo variance is small, we can have reasonable confidence in our maximization. Monte Carlo adjusted profile (MCAP) methods [43,44] provide methodology which reduces and evaluates the consequences of this Monte Carlo error. MCAP methods have been used in various recent investigations using particle filters [38,45–47]. Our revised manuscript includes MCAP confidence intervals for both Model 1 and Model 3 (S7 Text).

We conclude that suitable diagnostic methods are available for the POMP methods we employ, and some of them are demonstrated and discussed in our paper. We would like to do what we can, within the constraints of this paper, to help build awareness of these diagnostic methods and ways in which they can be useful. A full exposition of this topic is out of the scope of this paper, though some of these diagnostics are included in supplement material (S5 Text, S7 Text). To further help the reader in this regard, we have added additional information in the Model 1 calibration subsection, which includes citations to papers that discuss these diagnostics or have extensive supplements that demonstrate these diagnostic issues in action.

One change we made in the statistical analysis that led to larger model likelihoods was increasing the computational effort in the numerical maximization. While IF2 enables parameter estimation for a large class of models, the theoretic ability to maximize the likelihood depends on asymptotics in both the number of particles and the number of filtering iterations. Many Monte Carlo replications are then required to quantify and further reduce the error. The large increase in the log-likelihood for Model 1 (Table 2) can primarily be attributed to increasing the computational effort used to calibrate the model. This result highlights the importance of carefully determining the necessary computational effort needed to maximize model likelihoods and acting accordingly. In this case study, this was done by performing standard diagnostics for the IF2 and particle filter algorithms [9, 38, 41, 48]. Given the considerable computational costs of simulation-based algorithms, we find it useful to perform an initial assessment using hyperparameter values—such as the number of particles, filtering iterations, and replicates based on different parameter initializations—that enable relatively quick calculations. The insights obtained from this preliminary analysis help in accurately determining the amount of computation that is required to achieve reliable outcomes.

For example, in Figure 5, department Ouest exhibits substantial uncertainty from 2014 onwards, and this figure is on a log scale. Essentially, the inference suggests that 'anything can happen'. I would like to pinpoint the nature of this collapse in uncertainty. Identifiability issues might be at play, given the possibility of more estimated parameters than the incidence data can inform. Sometimes, we ask too much from the data. Observe the discrepancy in trends between the average behaviour and the uncertainty ribbons.
In summary, the authors should elaborate on the limitations of the proposed approach.

We agree with the referee that uncertainty quantification and evaluation of identifiability are fundamental tasks for model-based data analysis. In the revision, we have included profile confidence intervals on all parameters, except those taking separate values on each unit (S7 Text). For these unit-specific parameters, the spread of values across units provides an alternative measure of uncertainty. We find that all parameters are identifiable, though some are more tightly estimable than others.

We had not done this in the original submission since it was only tangentially relevant to the comparison with [1] that lies at the center of our paper. In particular, [1] did not present confidence intervals. However, these additional results are worthwhile extension to our analysis and we are grateful for the encouragement to include it.

Wide prediction intervals are not necessarily a sign of a problem. For example, nobody knew for sure whether cholera was banished from Haiti after it was declared eliminated in February 2022. Cholera reemerged in September 2022, but a reasonable model might be expected to cover the possiblity of this reemergence either happening or not happening, leading to wide prediction intervals. It is also critical to recognize that the simulations in Figure 5 are obtained from initial conditions, effectively making simulations at the end of the time series (January 2019) 8 year forecasts from the starting point in November 2010. The 95% confidence interval for Ouest in this figure is from $0 - 1094$ reported cases. This range may not be considered unreasonably large given the inherent stochasticity present in the dynamic system and all of the many possible outcomes of the outbreak from its initial state in 2010.

3. Conclusions

I find that the conclusions are somewhat disconnected from the introduction and abstract, which state that the paper presents a methodology to diagnose model misspecification, develop alternative models, and make computational improvements. It would improve readability to include a summary in this section. Specifically, link each contribution to a particular example. For instance, in the case of model 1, computational improvements increased the log-likelihood. In short, connect the findings more explicitly to the research question and stated goals.

We have added several paragraphs in the discussion to address this point. These paragraphs are provided below for reference.

Model diagnostics are a key tool for exposing unresolved model limitations and improving model fit. In our case study, we compared the three models from Lee et al. [1] to statistical benchmarks, revealing areas for improvement. For example, comparisons of Model 3 to a benchmark revealed its inadequacy in accounting for the post-hurricane increase in transmission, leading to a beneficial model refinement. When a mechanistic model is competitive with statistical benchmarks, we have a license to begin critical evaluation of its causal implications. If a model falls far behind simple benchmarks, there is likely to be substantial limitation in the data analysis that should be identified and remedied. In our case study, the re-calibrated version of Model 1 outperformed its benchmark, so we proceeded to examine causal implications. When doing so, we found that the fitted model provides a causal description of the dynamic system that is consistent with known features of the system, such as the importance of rainfall as a driver of cholera infection. The congruency between causal implications of the model and our belief about the dynamic system, coupled with a strong quantitative description of observed data relative to a benchmark, provides support for viewing the model as a plausible quantitative representation of the system under investigation.

When fitting a mechanistic model to a dynamic system, the complexity of the model warrants consideration. Mathematical models provide simplified representations of complex systems, with the simplicity serving both to facilitate scientific understanding and to enable statistical inference on unknown parameters. In our case study, employing deterministic dynamics in Model 2 was found to be an over-simplification by comparing model fit with benchmarks. Model 3 is distinct in that it is both stochastic and has a meta-population structure, making it challenging to draw likelihood-based inferences. In this paper, we demonstrated how this model class can be calibrated to incidence data using the innovative IBPF algorithm. One of only a few examples of fitting a nonlinear non-Gaussian meta-population model via maximum likelihood [9, 49], this case study exemplifies the algorithm's potential benefits and provides an example for future researchers on a possible approach to fitting a high-dimensional non-linear model.

Likelihood-based methods aid in determining an appropriate level of model complexity. Models fit to the same data can be compared using a criteria such as AIC. Nested model variations are particularly useful as they enable formal statistical testing of the nested features via likelihood ratio tests. Our case study demonstrated the examination of nested model features for all three models. Model 1 investigated a time-varying transmission rate; Model 2 assessed a phase-shift parameter in seasonal cholera peaks; Model 3 incorporated hurricane-related parameters.

Unmodeled features of a dynamic system can lead to spurious or misleading parameter estimates if the features substantially impact observed data. In deterministic models, features that cannot be explained by measurement error must be accounted for by the choice of parameters. For our case study, some of the parameter estimates for the deterministic Model 2 are implausible, such as the infinite immunity discussed above, and this may be explained by compensation for model misspecification. Incorporating demographic and environmental stochasticity into models can mitigate the impact of unmodeled features. Stochastic phenomena are not only arguably present in biological systems, but their inclusion in a model also allows observed data variations to be attributed to inherent uncertainty rather than to distorted parameter values. Models 1 and 3 suggest the presence of extra-demographic stochasticity [9, 20, 23], as evidenced by the confidence intervals for the corresponding parameter σ_{proc} *(S7 Text).*

If forecasts are an important component of a modeling task, the forecasts should be consistent with the available data, particularly at the most recently available time points. In our case study, we did this by simulating forward from the filtering distribution, as this procedure conditions latent variables on the available data. This type of forecasting, however, is not directly available using a deterministic model, where future dynamics are fully determined by initial conditions and parameter values. This can result in over-confident model forecasts [24]. Despite their limitations, deterministic models can offer valuable insights into dynamic systems [16]. In [1], the forecasts from the deterministic Model 2 were qualitatively more consistent with the observed disappearance of cholera than the stochastic models. In our case study, we found improvements to Models 1 and 3 that resulted in improved forecasts for these models.

Minor Comments:

1. In the author summary, this part is hard to follow: "and provides careful justification of valid conclusions from the fitted model. Objective measures are used to benchmark model fit; when these are combined with reproducibility, a framework emerges for continual improvement when revisiting the data and models." Please rephrase.

The previous statement has been changed to the following:

Our analysis presents methodology for diagnosing how well a model describes observed data. Using objective measures to assess model fit ensures that our evaluation is based on quantifiable criteria. Incorporating reproducibility into this assessment results in a framework that enables the validation or refinement of model based inferences when revisiting the data, facilitating scientific discovery.

2. Lines 1-8. Please add more citations.

Citations have been added.

3. Line 36. Can you be explicit about what the forecasts predicted? Did the models predict a rise in cases?

This statement has been modified to include more details. It now reads:

> *Despite their autonomy, the four independent teams obtained a consensus that an extensive nationwide vaccination campaign would be necessary to eliminate cholera from Haiti, estimating that a large number of cumulative cholera cases would be observed in the absense of additional vaccination efforts (Figure 3 and 4 of [1]). These forecasts are inconsistent with the prolonged period with no confirmed cholera cases between February, 2019 and September, 2022 [13].*

4. Line 42. Add hyphen: "Model-based conclusions".

A hyphen has been added for all instances of the phrase: "model-based".

5. Lines 67-78. Add more citations.

We have added a paragraph that includes a discussion and references on mechanistic vs associative modeling, which also addresses the concerns of Reviewer 1.

> *Mechanistic models representing biological phenomena are valuable for epidemiology and consequently for public health policy [14, 15]. More broadly, they have useful roles throughout biology, especially when combined with statistical methods that properly account for stochasticity and nonlinearity [16]. In some situations, modern machine learning methods can outperform mechanistic models on epidemiological forecasting tasks [17, 18]. The predictive skill of non-mechanistic models can reveal limitations in mechanistic models, but cannot readily replace the scientific understanding obtained by describing the biological dynamics of the system in a mathematical model [18, 19].*

6. Please add uncertainty intervals to the estimated parameters in Table 1. If necessary, consider splitting the table into two or including this additional information in the supplementary material. I suggest this update because there is an indication of unidentifiability when parameter estimates are fairly broad.

Due to the size and complexity of the table we have elected to put uncertainty estimates elsewhere (S7 Text). This additional supplement material document outlines how confidence intervals are obtained, and includes tables and figures that show parameter uncertainty estimates. In the caption for Table 1, we include a reference to this supplement material.

7. In line 159, it is stated that "$\upsilon^\star(t)$ is efficacy at time t since vaccination for adults" and then "single and double vaccine doses were modeled by changing the waning of protection; protection was modeled as equal between single and double dose until 52 weeks after vaccination, at which point the single dose becomes ineffective". I examined the reference from which this function is based but found only a numeric table. Please provide the equation of this function or a detailed description in the supplementary information. It would be beneficial for readers to understand how to model this complex feature.

The table you found is the function $\upsilon^\star(t)$, as that is what is used by each of their models. Lee et al [1] provide a smoothed version of this function in Figure 2 (B), but the actual function implemented

by each of their models (and consequently, our models) is the numeric table itself; in this sense, the smoothed function they show in Figure 2 (B) is an approximation of the true vaccination function that they use, which is provided in Table S4 in their supplement material.

According to Lee et al [1], this function was obtained by "fitting a log-linear weighted regression model to the raw data from a published meta-analysis on killed OCV efficacy against medically attended culter-confirmed cholera (figure 2, appendix 3 p 6) [50]". Aside from this statement and the table, they no additional details on how they came up with this function. Our revised manuscript clarifies this point

> *$\upsilon^\star(t)$ is efficacy at time t since vaccination for adults, a step-function represented in Table S4 of [1], ...*

> *8. Lines 209-211. In model 2, an incidence measurement is employed to configure a prevalence compartment. As the authors may know, initial values severely condition the dynamics of a model. Please explain this decision. Is it because that was the approach followed in the original formulation (Lee at al's paper)?*

Our starting point was the formulation of [1]. We did consider the effect of not calibrating initial values. Unsurprisingly, fitting initial values results in a quantitative improvement to model-fit, measured by AIC, but we also found that this had negligable qualitative effects on model-based conclusions. Importantly, the fitted model still falls short of the simple statistical benchmark used for comparison (S2 Text). We have included additional material in the supplement for Initialization Models that demonstrates this. On a nationally aggregated scale, the total number of initial infectious individuals in the fixed scenario was 36500, and 33480 when the initial states were estimated, which perhaps explains why calibrating initial parameters had little effect on qualitative conclusions.

> *9. Lines 245-247. Same comment as before. What's the justification for assuming incidence measurements as the basis for prevalence states? What are the risks?*

Our experience with mechanistic modeling reflects that of the reviewer: calibrating initial values often results in improved dynamics. As such, we would recommend calibrating initial values in most cases; here, doing so may constitute a major change from the initialization model used by Lee et al. [1]. Because one of our goals was to demonstrate that the models of [1] were alone not responsible for their poor forecasts, we elected to use the same initialization approach to the extent possible, as noted in the manuscript.

The goal of inferring initial prevalence is to reduce the number of parameters. The risk is that the form of this assumption could have substantial effects on the dynamics, and hence on the consequences of the analysis. In our supplement material (S5 Text), we discuss investigating conditional log-likelihoods of each observation in order to detect model misspecification. Here, we do not see clear evidence that fixing initial values in the same way that was done by [1] results in poor fits of the first few available observations. Specifically, we found that enforcing model dynamics for initial states worked well for many of the departments, but was problematic for departments where this resulted in zero counts in Infected, Asymptomatic, and Bacterial compartments, leading to the estimation of initial counts of Infected individuals in two departments (Grand'Anse and Nippes) (units 3 and 4, respectively). See Table 1 for estimates of initial values for these two units.

10. Line 262. "deterministic Model 2 is a degenerate case of a stochastic model". Please explain why or provide a reference.

The text has been modified to add additional clarity. The text now reads:

deterministic Model 2 is a special case of a Markov processes solving a stochastic differential equation in the limit as the noise parameter goes to zero.

11. Lines 260-274. Please add more citations.

We have added a citation to [41], which discusses POMP models and their implementation.

12. Lines 343-345. This sentence is key for this paper, but it's hard to follow: "The robust statistical conclusion is that a model which allows for change fits better than one which does not—we argue that a decreasing transmission rate is a plausible way to explain this, but the incidence data themselves do not provide enough information to pin down the mechanism". Please rephrase.

This suggestion is similar to one posed by Reviewer 1. We have made substantial edits to the paragraph that included this statement that clarifies our point:

If a mechanistic model including a feature (such as a representation of a mechanism, or the inclusion of a covariate) fits better than mechanistic models without that feature, and also has competitive fit compared to associative benchmarks, this may be taken as evidence supporting the scientific relevance of the feature. As for any analysis of observational data, we must be alert to the possibility of confounding. For a covariate, this shows up in a similar way to regression analysis: the covariate under investigation could be a proxy for some other unmodeled phenomenon or unmeasured covariate.

The statistical evidence of a trend in transmission rate in this model could be explained by any trending variable (such as hygiene improvements, or changes in population behavior), resulting in confounding from collinear covariates. Alternatively, it is possible that the negative trend observed in the incidence data could be attributed to a decreasing reporting rate rather than decreasing transmission rate. This could be formally tested by comparing models with either trend specification. We did not do this because evidence suggests that reporting rate was maintained or increased (Figure 1 of [10]). We instead argue that a decreasing transmission rate is a plausible way to explain the decrease in cases over time, as there is alternative evidence that supports this model [10–12]. It is not practical to test all remotely plausible model variations, yet a strongly supported conclusion should avoid ruling out untested hypotheses. The robust statistical conclusion for our analysis is that a model which allows for change fits better than one which does not, and a trend in transmission is a plausible way to do this.

13. Line 357. "Determining the necessary computational effort needed to maximize model likelihoods and acting accordingly" How do we determine the necessary computational effort?

We have added the following text to clarify this point:

The large increase in the log-likelihood for Model 1 (Table 2) can primarily be attributed to increasing the computational effort used to calibrate the model. This result highlights the importance of carefully determining the necessary computational effort needed to maximize model likelihoods and acting accordingly. In this case study, this was done by performing standard diagnostics for the IF2 and particle filter algorithms [9, 38, 41, 48]. Given the considerable computational costs of simulation-based algorithms, we find it useful to perform an initial assessment using hyperparameter values—such as the number of particles, filtering iterations, and replicates based on different parameter initializations—that enable relatively quick calculations. The insights obtained from this preliminary analysis help in accurately determining the amount of computation that is required to achieve reliable outcomes.

14. Please add the predicted intervals to Fig 4. I would like to see the effect of the log-normal measurement model.

We have added prediction intervals due to the error in the log-normal measurement model.

15. Lines 513-515. Please clarify the comparison between disaggregated models and a benchmark. Let's say I have spatial units 1 and 2, for which I have observations y1 and y2. Should I fit the disaggregated mechanistic model to y1 and y2 simultaneously (as usual) but keep a record of the individual log-likelihoods (log-lik y1 and log-lik y2). In parallel, fit the benchmark independently to y1 and y2, and then compare by log-likelihoods or information criteria by spatial unit.

Thank you for this suggestion. In general, a joint model for units 1 and 2 may not have well-defined individual log-likelihoods log-lik y1 and log-lik y2 that sum to the full likelihood. An exception arises in the case of likelihood evaluation by the block particle filter, in which conditinal log likelihoods for each block of units do sum to the total log likelihood estimate. If the block approximation is found to be appropriate [49,51] then fits can be compared against an independent benchmark for each block, and then summed to compare on the entire dataset.

We have added the following details to help clarify how the benchmarks for the spatially explicit models were created, and how to appropriately compare model likelihoods to these benchmarks:

To obtain a benchmark for models with a meta-population structure, we fit independent auto-regressive negative binomial models to each spatial unit. Under the assumption of independence, the log-likelihood of the benchmark on the entire collection of data can be obtained by summing up the log-likelihood for each independent model. In general, a spatially explicit model may not have well-defined individual log-likelihoods, and, in this case, comparisons to benchmarks must be made at the level of the joint model.

16. Line 528. Please add hyphen: "Model-based inference".

A hyphen has been added for all instances of the phrase: "model-based".

17. Lines 576-577. "We notice that the calibrated model favors higher levels of cholera transmission than what was typically observed in the incidence data (S5 Text)". After this fragment, please summarise in one or two sentences what it will be found in S5 Text.

We have expanded this paragraph to provide more details about the supplement material:

In the supplement material (S5 Text), we explore in more detail the process of model fitting and diagnostics for Model 3. Here we demonstrate that the model outperforms its benchmark model on the aggregate scale. However, when focusing on the spatial units with the highest incidence of cholera, Model 3 performs roughly the same as a simple benchmark. By comparing simulations from the fitted model to the filtering distribution, we see that the reconstructed latent states of the model favor higher levels of cholera transmission than what is typically observed in the incidence data. These results hint at the possibility of model mispecification, and warrant a degree of caution in interpreting the model's outputs.

18. Lines 599-601. "The decision not to do this partially explains the unsuccessful forecasts of Lee et al. [1]: their Table S7 shows that the subset of their simulations which were consistent with observing zero cases in 2019 also accurately predicted the prolonged absence of detected cholera". The fragment before the colon says that Lee was unsuccessful. However, the fragment after the colon says that was in part successful. Please clarify.

In their article, Lee et al. [1] only presented forecasts that were based on simulations from initial conditions. These forecasts were generally inconsistent with the absence of cholera cases from 2019-2022, and do not account for the particular relevance of recent data for forecasting cholera dynamics. In their supplement material, however, they provide a table (S7) in which they show that if they conditionally select only the simulations that were consistent with recent data (0 cases from 2019-2020), their forecasts predict cholera elimination. This second effort was only presented in the supplement material, but is more consistent with what we suggest is correct practice: forecasts of a system should start using latent states that are consistent with available data. We have expanded this argument in order to clarify this point:

Forecasts of a dynamic system should should be consistent with the available data. It is particularly important that forecasts are consistent with the most recent information available, as recent data is likely to be more relevant than older data. While this assertion may seem self-evident, it is not the case for deterministic models, for which the initial conditions together with the parameters are sufficient for forecasting, and so recent data may not be consistent with model trajectories. Epidemiological forecasts based on deterministic models are not uncommon in practice, despite their limitations [24]. Lee et al. [1] chose to obtain forecasts from all of their models by simulating forward from initial conditions, rather than conditioning forecasts based on the available data. This decision is possibly as a result of using a deterministic model, as forecasts from different models may only be considered comparable if they are obtained in the same way, which is most easily done be by simulating from initial conditions because Model 2 is deterministic.

In contrast, for non-deterministic Models 1 and 3, we obtain forecasts by simulating future values using latent states that are harmonious with the most recent data. This is done by simulating forward from latent states drawn at the last observation time (t_N) from the filtering distribution $f_{\boldsymbol{X}_N|\boldsymbol{Y}_{1:N}}(\boldsymbol{x}_N|\boldsymbol{y}^*_{1:N};\hat{\theta})$. The decision to obtain model forecasts from initial conditions partially explains the unsuccessful forecasts of Lee et al. [1]. Table S7 in their supplement material, which contains results that were not discussed in their main article, shows that the subset of their simulations with zero cholera cases from 2019-2020 also correspond with its disappearance until 2022. These results support our argument that forecasts should be made by ensuring the starting point for the forecast is consistent with available data.

19. Line 631. Since Model 1 accounts for infections at the national level, how are scenarios V1 and V2 handled?

We followed the same approach as Lee et al. [1], which was requiring that the same number of vaccinations are administered at the national level as they would have been in the spatially-explicit vaccination campaigns. The following sentence has been added to clarify this:

> *Because Model 1 only accounts for national level disease dynamics, the pre-determined department-specific vaccination campaigns are carried out by assuming the vaccines are administered in one week to the same number of individuals that would have obtained vaccines if explicitly administered to the specific departments. We refer readers to [1] and the accompanying supplement material for more details.*

References

1. Lee EC, Chao DL, Lemaitre JC, Matrajt L, Pasetto D, Perez-Saez J, et al. Achieving Coordinated National Immunity and Cholera Elimination in Haiti Through Vaccination: A Modelling Study. The Lancet Global Health. 2020;8(8):e1081–e1089.

2. Behrend MR, Basáẽz MG, Hamley JID, Porco TC, Stolk WA, Walker M, et al. Modelling for Policy: The Five Principles of the Neglected Tropical Diseases Modelling Consortium. PLoS Neglected Tropical Diseases. 2020;14(4):1–17.

3. Saltelli A, Bammer G, Bruno I, Charters E, Di Fiore M, Didier E, et al. Five Ways to Ensure that Models Serve Society: a Manifesto. Nature. 2020;582:428–484.

4. Donnelly CA, Boyd I, Campbell P, Craig C, Vallance P, Walport M, et al.. Four principles to make evidence synthesis more useful for policy; 2018.

5. Dahabreh IJ, Chan JA, Earley A, Moorthy D, Avendano EE, Trikalinos TA, et al.. Modeling and Simulation in the Context of Health Technology Assessment: Review of Existing Guidance, Future Research Needs, and Validity Assessment; 2017.

6. Egger M, Johnson L, Althaus C, Schöni A, Salanti G, Low N, et al. Developing WHO guidelines: time to formally include evidence from mathematical modelling studies. F1000Research. 2017;6.

7. Peñaloza Ramos MC, Barton P, Jowett S, Sutton AJ. A Systematic Review of Research Guidelines in Decision-Analytic Modeling. Value in Health. 2015;18(4):512–529.

8. Saltelli A. A short comment on statistical versus mathematical modelling. Nature communications. 2019;10(1):3870.

9. Li J, Ionides EL, King AA, Pascual M, Ning N. Machine Learning for Mechanistic Models of Metapopulation Dynamics. arxiv:231106702. 2023;.

10. Rebaudet S, Dély P, Boncy J, Henrys JH, Piarroux R. Toward Cholera Elimination, Haiti. Emerging Infectious Diseases. 2021;27(11):2932.

11. Rebaudet S, Bulit G, Gaudart J, Michel E, Gazin P, Evers C, et al. The Case-Area Targeted Rapid Response Strategy to Control Cholera in Haiti: A Four-Year Implementation Study. PLoS Neglected Tropical Diseases. 2019;13(4):e0007263.

12. Michel E, Gaudart J, Beaulieu S, Bulit G, Piarroux M, Boncy J, et al. Estimating Effectiveness of Case-Area Targeted Response Interventions Against Cholera in Haiti. Elife. 2019;8:e50243.

13. Trevisin C, Lemaitre JC, Mari L, Pasetto D, Gatto M, Rinaldo A. Epidemicity of Cholera Spread and the Fate of Infection Control Measures. Journal of the Royal Society Interface. 2022;19(188):20210844.

14. Lofgren ET, Halloran ME, Rivers CM, Drake JM, Porco TC, Lewis B, et al. Mathematical models: A key tool for outbreak response. Proceedings of the National Academy of Sciences of the USA. 2014;111(51):18095–18096.

15. McCabe R, Donnelly CA. Disease transmission and control modelling at the science–policy interface. Interface Focus. 2021;11(6):20210013.

16. May RM. Uses and abuses of mathematics in biology. science. 2004;303(5659):790–793.

17. Lau MS, Becker A, Madden W, Waller LA, Metcalf CJE, Grenfell BT. Comparing and linking machine learning and semi-mechanistic models for the predictability of endemic measles dynamics. PLoS computational biology. 2022;18(9):e1010251.

18. Baker RE, Pena JM, Jayamohan J, Jérusalem A. Mechanistic models versus machine learning, a fight worth fighting for the biological community? Biology letters. 2018;14(5):20170660.

19. Prosperi M, Guo Y, Sperrin M, Koopman JS, Min JS, He X, et al. Causal inference and counterfactual prediction in machine learning for actionable healthcare. Nature Machine Intelligence. 2020;2(7):369–375.

20. He D, Ionides EL, King AA. Plug-and-Play Inference for Disease Dynamics: Measles in Large and Small Towns as a Case Study. Journal of the Royal Society Interface. 2010;7:271–283.

21. Bretó C, He D, Ionides EL, King AA. Time Series Analysis via Mechanistic Models. Annals of Applied Statistics. 2009;3:319–348.

22. Lemaitre J, Pasetto D, Perez-Saez J, Sciarra C, Wamala JF, Rinaldo A. Rainfall as a Driver of Epidemic Cholera: Comparative Model Assessments of the Effect of Intra-Seasonal Precipitation Events. Acta Tropica. 2019;190:235–243.

23. Stocks T, Britton T, Höhle M. Model Selection and Parameter Estimation for Dynamic Epidemic Models via Iterated Filtering: Application to Rotavirus in Germany. Biostatistics. 2020;21(3):400–416.

24. King AA, Domenech de Cellès M, Magpantay FM, Rohani P. Avoidable Errors in the Modelling of Outbreaks of Emerging Pathogens, with Special Reference to Ebola. Proceedings of the Royal Society B: Biological Sciences. 2015;282(1806):20150347.

25. Kirpich A, Weppelmann TA, Yang Y, Morris Jr JG, Longini Jr IM. Controlling cholera in the Ouest Department of Haiti using oral vaccines. PLOS Neglected Tropical Diseases. 2017;11(4):e0005482.

26. Pasetto D, Finger F, Camacho A, Grandesso F, Cohuet S, Lemaitre JC, et al. Near real-time forecasting for cholera decision making in Haiti after Hurricane Matthew. PLOS Computational Biology. 2018;14(5):1–22. doi:10.1371/journal.pcbi.1006127.

27. Mukandavire Z, Smith DL, Morris Jr JG. Cholera in Haiti: reproductive numbers and vaccination coverage estimates. Scientific reports. 2013;3(1):997.

28. Kirpich A, Weppelmann TA, Yang Y, Ali A, Morris JG Jr, Longini IM. Cholera Transmission in Ouest Department of Haiti: Dynamic Modeling and the Future of the Epidemic. PLOS Neglected Tropical Diseases. 2015;9(10):1–12. doi:10.1371/journal.pntd.0004153.

29. Lewnard JA, Antillón M, Gonsalves G, Miller AM, Ko AI, Pitzer VE. Strategies to prevent cholera introduction during international personnel deployments: a computational modeling analysis based on the 2010 Haiti outbreak. PLoS medicine. 2016;13(1):e1001947.

30. Kunkel A, Lewnard JA, Pitzer VE, Cohen T. Antimicrobial resistance risks of cholera prophylaxis for United Nations peacekeepers. Antimicrobial agents and chemotherapy. 2017;61(8):10–1128.

31. Mukandavire Z, Morris Jr JG. Modeling the epidemiology of cholera to prevent disease transmission in developing countries. Microbiology spectrum. 2015;3(3):10–1128.

32. Sallah K, Giorgi R, Bengtsson L, Lu X, Wetter E, Adrien P, et al. Mathematical Models for Predicting Human Mobility in the Context of Infectious Disease Spread: Introducing the Impedance Model. International Journal of Health Geographics. 2017;16(1):1–11.

33. Azman AS, Luquero FJ, Rodrigues A, Palma PP, Grais RF, Banga CN, et al. Urban cholera transmission hotspots and their implications for reactive vaccination: evidence from Bissau city, Guinea bissau. PLoS neglected tropical diseases. 2012;6(11):e1901.

34. Azman AS, Luquero FJ, Ciglenecki I, Grais RF, Sack DA, Lessler J. The impact of a one-dose versus two-dose oral cholera vaccine regimen in outbreak settings: A modeling study. PLoS Medicine. 2015;12(8):e1001867.

35. Andrieu C, Doucet A, Holenstein R. Particle Markov chain Monte Carlo methods. Journal of the Royal Statistical Society, Series B (Statistical Methodology). 2010;72:269–342.

36. Fox SJ, Lachmann M, Tec M, Pasco R, Woody S, Du Z, et al. Real-time pandemic surveillance using hospital admissions and mobility data. Proceedings of the National Academy of Sciences of the USA. 2022;119(7):e2111870119.

37. Molodecky NA, Jafari H, Safdar RM, Ahmed JA, Mahamud A, Bandyopadhyay AS, et al. Modelling the spread of serotype-2 vaccine derived-poliovirus outbreak in Pakistan and Afghanistan to inform outbreak control strategies in the context of the COVID-19 pandemic. Vaccine. 2023;41:A93–A104.

38. Pons-Salort M, Grassly NC. Serotype-Specific Immunity Explains the Incidence of Diseases Caused by Human Enteroviruses. Science. 2018;361(6404):800–803.

39. Subramanian R, Romeo-Aznar V, Ionides E, Codeço CT, Pascual M. Predicting Re-Emergence Times of Dengue Epidemics at Low Reproductive Numbers: DENV1 in Rio de Janeiro, 1986–1990. Journal of the Royal Society Interface. 2020;17(167):20200273.

40. Coulson T, Rohani P, Pascual M. Skeletons, noise and population growth: The end of an old debate? Trends in Ecology and Evolution. 2004;19:359–364.

41. King AA, Nguyen D, Ionides EL. Statistical Inference for Partially Observed Markov Processes via the R Package pomp. Journal of Statistical Software. 2016;69:1–43.

42. Gneiting T, Raftery AE. Strictly Proper Scoring Rules, Prediction, and Estimation. Journal of the American Statistical Association. 2007;102(477):359–378.

43. Ionides EL, Breto C, Park J, Smith RA, King AA. Monte Carlo Profile Confidence Intervals for Dynamic Systems. Journal of the Royal Society Interface. 2017;14:1–10.

44. Ning N, Ionides EL, Ritov Y. Scalable Monte Carlo Inference and Rescaled Local Asymptotic Normality. Bernoulli. 2021;pre-published online.

45. Subramanian R, He Q, Pascual M. Quantifying Asymptomatic Infection and Transmission of COVID-19 in New York City using Observed Cases, Serology, and Testing Capacity. Proceedings of the National Academy of Sciences of the USA. 2021;118(9).

46. Ali ST, Lau YC, Shan S, Ryu S, Du Z, Wang L, et al. Prediction of upcoming global infection burden of influenza seasons after relaxation of public health and social measures during the COVID-19 pandemic: a modelling study. The Lancet Global Health. 2022;10(11):e1612–e1622.

47. Simpson MJ, Maclaren OJ. Profile-wise analysis: a profile likelihood-based workflow for identifiability analysis, estimation, and prediction with mechanistic mathematical models. PLoS Computational Biology. 2023;19(9):e1011515.

48. Laneri K, Bhadra A, Ionides EL, Bouma M, Yadav R, Dhiman R, et al. Forcing Versus Feedback: Epidemic Malaria and Monsoon Rains in NW India. PLoS Computational Biology. 2010;6:e1000898.

49. Ionides EL, Ning N, Wheeler J. An Iterated Block Particle Filter for Inference on Coupled Dynamic Systems with Shared and Unit-Specific Parameters. Statistica Sinica, pre-published online. 2022;.

50. Bi Q, Ferreras E, Pezzoli L, Legros D, Ivers LC, Date K, et al. Protection against cholera from killed whole-cell oral cholera vaccines: a systematic review and meta-analysis. The Lancet Infectious Diseases. 2017;17(10):1080–1088.

51. Ionides EL, Asfaw K, Park J, King AA. Bagged filters for partially observed interacting systems. Journal of the American Statistical Association. 2021;pre-published online. doi:10.1080/01621459.2021.1974867.

S2 Text Initialization models. Additional details of the initialization model that were used for Models 1–3.

S5 Text Calibrating Model 3. Additional details on the procedures for fitting and diagnosing Model 3.

S7 Text Confidence Intervals Tables and figures describing 95% confidence intervals for estimated parameters.
