# Supplementary material for: Informing policy via dynamic models: Cholera in Haiti
Source: PLoS Comput Biol. 2024 Apr 29;20(4):e1012032. doi: 10.1371/journal.pcbi.1012032 (PMC11081515; doi:10.1371/journal.pcbi.1012032)
Supplement: Attachment — Submitted filename: plos_comb_bio_review_2023.docx [file pcbi.1012032.s012.docx]

The authors’ manuscript aims to explain how public health decisions can best be informed by combining an understanding of the underlying pathogen transmission dynamics with epidemiological data. In particular, the authors make a number of recommendations of best practices that modellers can adopt when using mechanistic models to make statistical inferences from time series data. To make their recommendations more concrete, they perform a re-analysis of cholera incidence data from Haiti spanning the years 2014-2019. These data were previously analysed in a modelling study performed by a separate team of scientists, published in Lee et al. (2020), see Ref. 1. Using refinements of three models from Lee et al., the authors demonstrate a number of ways in which the analysis could have been improved. Furthermore, the authors present differences between the findings of their reanalysis and the original analysis of Lee et al., for instance in the predicted probability of eliminating cholera from Haiti (see Figure 8).

My most substantial concern is with the framing of the paper, and whether that might mislead potential readers. To me, the paper serves more of an educational purpose, presenting ideas on how to best use mathematical models to gain insight from time series data. While the case study into cholera in Haiti is methodologically sound, it does not seem to be the main focus of the manuscript. To such an extent that, in the discussion, the only comment to on the Haiti analysis is that the authors “used the same data and models, and even much of the same code, as Lee et al. [1], and yet ended up with drastically different conclusions.”

This major concern can be resolved in two ways, both of which seem acceptable to me, either a) reframe the paper as a more general series of recommendations for how to better perform statistical inference on time series data using mechanistic models (including a longer introduction and discussion of existing methodological deficiencies) or b) perform a more in-depth analysis and commentary on cholera in Haiti (some of which has already been done but is buried in supplementary information files that are only briefly mentioned in the main text, e.g. S5).

I found the methodology used to be well-suited to addressing the aims of the paper.

Overall, I found the paper interesting and informative, but a little unfocused. I expect with some heavy rewriting in places (especially the introduction and discussion), the paper will be acceptable for publication in PLoS Computational Biology. I do not anticipate the authors having to perform additional computational analysis.

Abstract

1. The authors state they “develop data analysis strategies leading to improved statistical fit.” Its not clear to me exactly what novel strategies have been developed, it seems the authors exclusively use pre-existing techniques from the literature. This is fine, if the focus of the paper is either a) pedagogical or b) on understanding a specific disease system (cholera in Haiti). If any methods are novel, this should be more clearly stated.

Introduction

1. To make clear that the issues being addressed by this paper go beyond one modelling study of Haiti, the authors should provide examples and references to support the (accurate in my opinion) statement about, “common modeling decisions that may not provide an adequate statistical explanation of the data”.
2. Given the focus of the paper on how to better perform inference, explicitly commenting on what are the “existing guidelines for creating models to inform policy [4, 5]”. It would then also be helpful in the discussion to comment on how the recommendations of this study extend these pre-existing guidelines.

Methods

3. Fig 2. Model parameters. I think this should be “Table 1”?
4. “and z ∈ 1:Z describes hypothetical vaccination programs” It would help to give an example of the different vaccine programs here. E.g. does this mean the number of doses the individual has received, or differences in the vaccine administered?
5. Eq 6 and line 1: I think the notation for the asymptomatic fraction might be clearer and more consistent with the other models if you used $f_z(t)$ instead of $\vartheta_z(t)$. In fact, this is the notation used in the table in Figure 2.
6. Line 198: Reference for where value of v_rate comes from.
7. Usually, vaccine efficacy = 1 implies the vaccine completely blocks transmission and 0 implies no effect. In model 2 the definition of vaccine efficacy (lines 200-205 and Eq 14) implies the opposite. I suggest re-parameterising the model, or renaming the variable. For what its worth, models 1 and 3 seem to follow the usual convention.
8. I found the commentary on comparing fitted mechanistic models to a (statistical) benchmark model to be informative and helpful. I was wondering if the authors had any comments on whether (or not) to directly compare the AIC values of the different fitted models directly. Am I right in thinking this should be possible for models 2 and 3 given (I think) they are fitted to the same data? Model 1 is fitted to a different data set, which I understand makes such a comparison impossible.
9. Lines 308-309: Isn’t this a log-linear trend in the transmission rate?
10. While the data support a linear trend at the 95% confidence level, it's worth commenting on the magnitude of the trend, e.g. the total reduction in β(t) over the period of study.
11. I might have misunderstood it slightly, but I don’t entirely follow the reasoning of lines 340-346. It seems that two different claims are being made: A) the model can’t identify which mechanism underlies a trend in β(t) and A) the model can’t definitively state there is a trend in β(t) compared with some other time-varying parameter (e.g. reporting probability). For model 1, I agree with the authors that claims A and B are valid. What I take issue with is the final statement, which seems to be stronger and apply to both claims and mechanistic models more generally: “we argue that a decreasing transmission rate is a plausible way to explain this, but the incidence data themselves do not provide enough information to pin down the mechanism.” I agree that the “incidence data themselves” (which I take to imply "without additional covariates data") make claim A valid regardless of the model. However, I don’t think claim B holds regardless of the model. By changing model 1 to include time variation in the reporting rate I don’t see *a priori* why the “the incidence data themselves” might not provide enough information to distinguish between time variation in transmission compared with time variation in reporting *without the need for additional data.* The deficiency is in model 1 rather than the information content of the data. I suggest the authors rework the paragraph to make the reasoning clearer.
12. Lines 53-56: I push back against this point. The models assume an exponential distribution for vaccine-derived immunity. Assuming a mean duration of 10 years, the proportion of individuals who remain immune 9 years after vaccination is $e^{-9/10}\approx0.41$. Even for much larger durations of immunity a non-negligible fraction of the population will lose immunity after 9 years, e.g. $e^{-9/100}\approx0.91$. I therefore don’t expect values for the duration of immunity around 10 years to “effectively result in the same model dynamics”.

Discussion

13. In contrast with the introduction, which almost exclusively focuses on cholera in Haiti, the discussion only mentions it once, briefly: “We used the same data and models, and even much of the same code, as Lee et al. [1], and yet ended up with drastically different conclusions.” It would help the reader to have those discrepancies summarised.
14. It would be helpful to summarise the recommendations the authors make attempting to fit mechanistic models to time series data (even as a list).
15. Along similar lines, it would be helpful to present a more concise summary of what the “more accurate policy evaluations” found by using the approaches outlined in this study.

Typos:

1. Eq. 1: Is there a missing star on $y_{1:N}$ on the right-hand-side?
2. Line 146: delete “the” before describing
